# Tertiary lymphoid structure-related score as a predictor for survival prognosis and immunotherapy response in head and neck squamous cell carcinoma

**DOI:** 10.3389/fimmu.2024.1483497

**Published:** 2024-10-18

**Authors:** Fan Wu, Haotian Cao, Siqi Ren, Jiaying Wu, Xin Liu, Qunxing Li, Qiuping Xu, Jiali Chen, Ruixin Wang, Suling Chen, Shijia Kuang, Binbin Xia, Yanyan Li, Liansheng Wang, Jintao Li, Bowen Li, Jinsong Li, Tianjun Lan

**Affiliations:** ^1^ Department of Oral and Maxillofacial Surgery, Sun Yat-sen Memorial Hospital of Sun Yat-Sen University, Guangzhou, Guangdong, China; ^2^ Guangdong Provincial Key Laboratory of Malignant Tumor Epigenetics and Gene Regulation, Guangdong-Hong Kong Joint Laboratory for RNA Medicine, Medical Research Center, Sun Yat-sen Memorial Hospital, Sun Yat-sen University, Guangzhou, Guangdong, China; ^3^ Department of Stomatology, Affiliated Hospital of Hangzhou Normal University, Hangzhou, Zhejiang, China; ^4^ Department of Stomatology, Shunde Hospital, Southern Medical University (The First People’s Hospital of Shunde), Foshan, Guangdong, China; ^5^ Medical Research Center, Sun Yat-sen Memorial Hospital, Sun Yat-sen University, Guangzhou, Guangdong, China

**Keywords:** tertiary lymphoid structure, head and neck squamous cell carcinoma, TLSRGs, TLSscore, immunotherapy

## Abstract

**Background:**

Substantial studies reveal that tertiary lymphoid structure (TLS) correlate with prognosis and immunotherapy response in various types of cancers. However, the predictive value of TLS, the specific immune cell subtype within TLS and their anti-tumor mechanisms remain unclear.

**Methods:**

Based on 23 TLS-related genes (TLSRGs), we utilized bioinformatics methods to construct a scoring system, named TLSscore. By integrating RNA and single-cell sequencing data, we assessed the utility of TLSscore in head and neck squamous cell carcinoma (HNSCC). Flow cytometric sorting was used to isolate specific T cells subtypes, *in vivo* and *in vitro* experiments were conducted to demonstrate its anti-tumor effects.

**Results:**

The TLSscore model was constructed and specific TLSscore-genes were found to consistently align with the spatial location of TLS. TLSscore has proven to be a robust predictive model for predicting survival prognosis, immune cell infiltration, somatic mutation and immunotherapy response. Notably, a specific PD1^+^CXCL13^+^CD8^+^T cell subtype was identified within TLS. Both *in vivo* and *in vitro* experiments demonstrated that PD1^+^CXCL13^+^CD8^+^T cell might represent a functional cell subtype exerting anti-tumor effects during the process of immune surveillance.

**Conclusions:**

Our study presents a predictive model for TLS, which can evaluate its presence and predicts survival prognosis and immunotherapy response in HNSSC patients. Additionally, we identify a specific subtype of T cells that might elucidate the mechanism of TLS function in anti-tumor activities. This T cell subtype holds the potential to be a prognostic marker and a target for adoptive cell therapy (ACT) in the future.

## 1 Introduction

Head and neck carcinoma (HNC) has become the 7th most prevalent malignant tumor globally (1, 2). Among these, head and neck squamous cell carcinoma (HNSCC) is the most common pathological type associated with a poor prognosis (3). Local recurrence and cervical node metastasis are two main causes of functional sequelae and mortality (4, 5). For patients suffering recurrent or metastatic squamous cell carcinoma who could not bear the surgery, the EXTREME regime (cisplatin, 5-fluorouracil and cetuximab) has emerged as one of the most commonly utilized chemotherapy strategies and is now considered the standard first-line treatment (6–8). Nonetheless, chemoresistance and toxicity not only lead to treatment failure but also give rise to various adverse effects (9, 10). Recent research indicates that immune evasion occurs when tumor cells leverage immune checkpoints to suppress T cell activity. Immunotherapies, particularly immune checkpoint inhibitors (ICIs) targeting the programmed cell-death protein 1 (PD-1) and programmed cell-death 1 ligand 1 (PD-L1), have achieved remarkable progress in the treatment of numerous cancers (11–13). Despite the obvious improvement in cancer prognosis, only 10% to 20% of HNSCC patients experience benefits from this treatment (14, 15). In addition, no biomarkers have been reported to exhibit strong predictive capacity for determining the response to immunotherapy (16, 17). Therefore, novel prognostic indicators accurately predicating and evaluating the response to ICIs in HNSCC are urgently needed.

Given the absence of a reliable indicator for assessing the efficacy of ICIs, the tertiary lymphoid structure (TLS) has been reported to hold potential prognostic value. TLS is an ectopic lymphoid organ containing T and B lymphocyte colonies as well as high endothelial venules, which develops in non-lymphoid tissues in response to chronic inflammation or tumors and plays a critical role in facilitating antigen presentation and promoting T and B cell activation (18). Numerous studies have investigated the correlation between TLS and clinical benefits of tumor patients. Consistent with recent studies across various tumor types, our previous investigation also demonstrated that the presence of TLS in HNSCC is associated with an improved prognosis (19–23). Furthermore, the latest research had confirmed that TLS is connected to a high response rate to immunotherapy with ICIs, which suggest that TLS is a crucial predictive factor of immunotherapy (24–26). However, the mechanisms underlying the antitumor responses of immune cells within TLS remain unclear.

In this study, we downloaded the mRNA sequencing data of HNSCC patients from TCGA database and utilized the bioinformatics methods to construct a scoring system named TLSscore based on TLSs-related genes. The prognosis analysis, immune cell infiltration pattern, somatic mutation and tumor immunogenicity analysis were further analyzed using the TLSscore model. It was found that genes of the TLSscore model have the capability to predict the clinical outcome of ICIs and the overall survival rate of patients with HNSCC. Finally, single-cell sequencing was performed to analyze the correlation between TLSscore and immunotherapy. A specific CD8^+^T cell subgroup within the TLS was found to exhibit potent anti-tumor capabilities. These findings elucidate the mechanism of TLS in immunotherapy and its anti-tumor effects.

## 2 Methods

### 2.1 Data acquisition

The transcription data of HNSCC was extracted from public databases, including Gene Expression Omnibus (GEO) and the Cancer Genome Atlas (TCGA). Three cohorts with HNSCC (GSE41613, GSE42743 and TCGA-HNSCC) comprising 671 patients with follow-up information were collected for analysis (**Supplementary Table S1**). GSE41613 and GSE42743 were microarray data from the Affymetrix Human Genome U133 Plus 2.0 Array platform. RNA sequencing (FPKM value) data, somatic mutation data, SCNAs and clinical data from TCGA were obtained from UCSC Xena. FPKM values were transformed into transcripts per kilobase million (TPM) values. To reduce non-biological technical biases among cohorts, the “ComBat” algorithm in the sva package was applied to correct batch effects. Additionally, three single-cell RNA sequencing data sets were obtained. GSE172577, published by our lab, included 6 samples from HNSCC patients, with 3 samples confirmed as TLS-positive via multiple immunohistochemistry (mIHC) and the rest are TLS-negative. The GSE195832 cohort comprised four patients with advanced-stage HNSCC who underwent anti-PD-1 therapy using nivolumab. The GSE123813 data contained 53,029 cells from 11 patients with advanced basal cell carcinoma (BCC) before and after anti-PD-1 treatment. No chemotherapy or radiotherapy was administered prior to these treatments.

### 2.2 Unsupervised clustering for TLSRGs

The 23 TLSRGs were obtained from a previous published study (27). Expression data for these 23 TLSRGs were extracted from three integrated HNSCC datasets to identify different TLSRG modification patterns. These 23 TLSRGs comprised 6 chemokines (CCL18, CCL19, CCL20, CCL21, CXCL9, CXCL13), 2 chemokine receptors (CCR5, CXCR3), 2 cytokines (IL10, CSF2), 4 transcription factors (CD200, GFI1, IRF4, STAT5A), 4 co-stimulatory molecules (ICOS, CD38, CD40, SH2D1A), 2 inhibitory receptors (TIGIT, PDCD1), 2 cytokine receptors (IL2RA, IL1R2) and 1 ECM-associated molecule (FBLN7). The ConsensusClusterPlus package was used to determine the number of clusters, with the optimal number selected based on the proportion of ambiguous clustering (PAC) score. To ensure classification stability, these steps were repeated 1000 times.

### 2.3 Immune cell infiltration and functional analysis

In this study, we used the immunedeconv package to quantify the proportions of immune cells in HNSCC samples, as described in a previous study (28). We also employed the ssGSEA algorithm to quantify the relative abundance of each cell infiltration in the HNSCC tumor microenvironment (TME). To estimate and quantify tumor purity, we used the ESTIMATE method, which calculates three scores (Immune score, Stromal score and Estimate score) representing the proportion of immune or stromal components in each patient. For pathway activity analysis between the TLSRG modification patterns, we used the GSVA package. Gene sets were obtained from the MSigDB database for GSVA analysis (gene set “c2.cp.kegg.v2022.1”). Additionally, we employed the clusterProfiler package (version 4.0.2) to conduct GO and KEGG analysis for the 23 TLSRGs. Data with a *P* value adjusted by the Benjamini and Hochberg method less than 0.05 were considered statistically significant.

### 2.4 Construction of the TLSRGs signature

To quantify the TLS modification for individual patients, we established a TLS scoring system, termed TLSscore, via principal component analysis. Firstly, we used the limma R package to identify differentially expressed genes (DEGs) between the TLSRGs modification patterns. Genes meeting the criteria of adjusted *p* value < 0.01 were considered as DEGs. Secondly, we divided the patients with HNSCC into clusters using an unsupervised clustering method on the identified DEGs. The proportion of ambiguous clustering (PAC) score was used to define the number of gene clusters and assess their stability. We then analyzed the TME cell infiltration characteristics and overall survival of DEGs based on the consensus clusters. Thirdly, we selected the DEGs between different consensus clusters and assessed the prognostic value of each gene using univariate Cox regression (*p* < 0.05). Subsequently, we conducted PCA analysis on the resultant prognostic genes to establish a TLS-related gene signature, selecting principal components 1 and 2 as the signature score. This approach focuses the TLSscore on the largest blocks of well-correlated (or anti-correlated) genes. Finally, we defined the TLSscore using the formula: TLSscore = ∑LPC1i - PC2i), where i represents the expression of prognostic TLS phenotype-related genes. To stratify patients into two distinct prognostic groups (TLSscore high and low groups), we used the “maxstat” R package to identify the best cut-off value.

### 2.5 Somatic mutation and tumor immunogenicity analysis

To investigate the relationship between TLSscore and tumor immunogenicity markers, we compared the expression levels of these markers between the TLSscore high and low groups. The tumor immunogenicity markers used in this study included tumor mutation burden (TMB), intratumor heterogeneity (ITH), homologous recombination deficiency (HRD) and aneuploidy. Furthermore, we downloaded somatic mutation and copy number variation (CNV) data of TCGA-HNSCC from the UCSC Xena database. We used the “maftools” R package to visualize the mutation landscape of the HNSCC samples. Significant gene deletions and amplifications were identified using GISTIC_2.0.

### 2.6 Immunotherapy response prediction

Based on published research, checkpoint-related genes, MHC I molecules genes and MHC II molecules genes are associated with the outcome of immunotherapy. Therefore, we predicted the immunotherapy response for each HNSCC sample based on the expression levels of these three gene sets. Additionally, we utilized The Cancer Immunome Atlas (TCIA) database (https://tcia.at/) to investigate immunotherapy sensitivity. We also employed the Tracking Tumor Immunophenotype (TIP) database to visualize the activity of anti-cancer immunity and the extent of tumor-infiltrating immune cells across the seven-step cancer-immunity cycles.

### 2.7 Single cell RNA sequencing analysis for TLSscore

In this study, we reanalyzed three single-cell transcriptomics datasets (GSE172577, GSE195832, and GSE123813) to investigate the potential role of TLSscore in HNSCC. The Seurat and Harmony R toolkits were employed to process the single-cell transcriptomics data. We applied the same quality control criteria to preprocess the GSE172577 and GSE195832 datasets, removing cells with UMI counts above 50,000 or fewer than 1,000, detected genes above 5,000 or fewer than 300, fraction of hemoglobin genes > 5%, and fraction of mitochondrial genes > 15%. Additionally, we used the DoubletFinder package to identify doublets. Subsequently, Harmony was used to integrate these samples. The NormalizeData function in the Seurat package was utilized to normalize the UMI counts, and 2,000 highly variable genes were identified via the FindVariableFeatures function. The RunPCA, FindNeighbors, FindClusters, and RunUMAP functions were run under default parameters unless specified otherwise. We selected the top 20 principal components for further UMAP visualization. The AddModuleScore function was employed to calculate the TLSscore. Since the 53,029 cells in GSE123813 were confirmed being certain cell types, we did not redefine these cells in this study. The data processing steps, including NormalizeData, FindVariableFeatures, RunPCA, FindNeighbors, FindClusters, and RunUMAP, were also applied to analyze the GSE123813 dataset.

### 2.8 Investigation for the role of TLSRGs in pan-cancer analysis

The multi-omics data of pan-cancer cohorts were obtained from the Genomic Data Commons (GDC) Pan-Cancer dataset in UCSC Xena. This dataset includes 33 different cancers, such as adrenocortical carcinoma (ACC), bladder urothelial carcinoma (BLCA), breast cancer (BRCA), cholangiocarcinoma (CHOL), colon adenocarcinoma (COAD), cervical squamous cell carcinoma and endocervical adenocarcinoma (CESC), lymphoid neoplasm diffuse large B-cell lymphoma (DLBC), esophageal carcinoma (ESCA), glioblastoma multiforme (GBM), head and neck squamous carcinoma (HNSC), brain lower grade glioma (LGG), lung adenocarcinoma (LUAD), lung squamous cell carcinoma (LUSC), liver hepatocellular carcinoma (LIHC), acute myeloid leukemia (LAML), kidney renal clear cell carcinoma (KIRC), kidney renal papillary cell carcinoma (KIRP), kidney chromophobe (KICH), mesothelioma (MESO), ovarian serous cystadenocarcinoma (OV), prostate adenocarcinoma (PRAD), pheochromocytoma and paraganglioma (PCPG), pancreatic adenocarcinoma (PAAD), rectum adenocarcinoma (READ), uterine carcinosarcoma (UCS), uterine corpus endometrial carcinoma (UCEC), uveal melanoma (UVM), stomach adenocarcinoma (STAD), skin cutaneous melanoma (SKCM), sarcoma (SARC), thyroid carcinoma (THCA), testicular germ cell tumors (TGCT) and thymoma (THYM). We used the edgeR package to identify differentially expressed genes (DEGs) between tumor and normal samples in this study. Genes meeting the criteria of an adjusted p-value < 0.05 were considered as DEGs. Subsequently, we investigated the prognostic characteristics of TLSRGs among the 33 cancer types using the survival package. Next, based on the somatic mutation data (SNV data), we calculated the single nucleotide variant (SNV) mutation frequency of the 23 TLSRGs across the 33 cancer types. Finally, we explored the relationship between copy number variation (CNV) and the expression level of each TLSRG using Pearson’s correlation.

### 2.9 Spatial transcription analysis

To investigate the spatial relationship between TLSscore and TLSs, we obtained spatial transcriptomics sequencing data (GSE175540) from the GEO database. We selected a TLS-positive FFPE renal cell cancer sample and a TLS-negative FFPE sample for analysis. We used the AddModuleScore function to calculate the TLSscore and applied Wilcox’s test to detect differences in TLSscore between TLS-positive and TLS-negative samples.

### 2.10 Multiplex immunohistochemistry (mIHC) staining

The multiplex immunohistochemistry (mIHC) analysis was conducted meticulously using the PANOVUE Manual IHC Kit (#PPK007100100, China). The primary antibodies used in this study included CD8 (1:200, #85336, CST), CD20 (1:200, #48750, CST), CXCL13 (1:200, #ab246518, abcam) and PD1 (1:500, #ab237728, abcam). The protocol involved several carefully orchestrated steps. Initially, tumor tissue sections were dewaxed by air-drying at 60°C for 1 hour, followed by a 30-minute xylene treatment. Subsequently, the sections underwent rehydration through a graded alcohol series. Antigen retrieval was achieved using EDTA buffer (pH 9.0) in a microwave-assisted process. Primary antibodies were then applied for incubation. To amplify the tyrosine signals, the TSA PANOVUE kit was used. The antigen retrieval, antibody incubation, and TSA amplification steps were repeated iteratively for each subsequent antibody in the panel. Slides were scanned using the Vectra scanner (Akoya, USA) for visualization and analysis of the stained sections. The resultant images were analyzed using the inForm Advanced Image Analysis software (inForm v2.3.0; PerkinElmer), providing a comprehensive and detailed assessment of the immunohistochemical markers.

### 2.11 T-cell activation and cell sorting

Peripheral blood mononuclear cells (PBMCs) were isolated using a standard procedure. Firstly, 5 ml of fresh peripheral blood was collected in EDTA-containing anticoagulant tubes before treatment initiation. The blood sample was then layered over Lymphoprep™ solution (#07801, STEMCELL) to separate PBMCs from other blood components. After centrifugation, the lymphocyte layer was transferred to a new 50 ml tube and washed with phosphate-buffered saline (PBS) to remove contaminants. The lymphocytes were incubated with a red blood cell lysis solution on ice for 10 minutes to remove erythrocytes. Subsequently, the cells were resuspended in sorting buffer (PBS with 2% fetal bovine serum) for downstream applications. 2 x 10^6^ naive T cells were activated in complete RPMI-1640 medium supplemented with 10% FBS, CD3/CD28 MicroBeads (#130-050-101 and 130-093-247, Miltenyi Biotec) and 2 ng/ml TGFβ-1 (#100-21, Propretech), following the manufacturer’s guidelines. Before sorting, the cells were blocked with Fc Receptor Blocking Solution (#422301, Biolegend) to minimize non-specific binding. Finally, activated T cells were sorted into PD1^+^CD39^+^CD103^+^CD8^+^T cell and PD1^+^CD39^-^CD103^-^CD8^+^T cell populations using flow cytometry and subsequent analysis of CXCL13 expression (PE anti-PD1, #12-9969-42, eBioscience; FITC anti-CD39, 328206, Biolegend; BV421 anti-CD103, 350214, Biolegend; APC anti-CXCL13, #MA523629, Invitrogen).

### 2.12 Cell culture

HNSCC cell lines Cal-27 and SAS were purchased from the American Type Culture Collection (ATCC) and the Chinese Academy of Sciences, respectively. Both cell lines were cultured in DMEM supplemented with 10% fetal bovine serum and maintained at 37°C with 5% CO2.

### 2.13 Cytotoxic experiments

SAS and CAL27 cells at 2 x 10^3^ per well were seeded into 96-well plates to establish a controlled environment for coculture assays. PD1^+^CD39^+^CD103^+^CD8^+^T cells and PD1^+^CD39^-^CD103^-^CD8^+^T cells were then added to these cocultures at effector-to-target (E:T) ratios of 1:1, 5:1, 10:1, 25:1 and 50:1. Importantly, no exogenous cytokines were added to ensure that the observed effects were solely due to direct interactions between T cells and cancer cells. Cytolytic activity was assessed using the LDH-Glo™ Cytotoxicity Assay (Promega), following the manufacturer’s instructions. Specific lysis of target cells was quantified by calculating the mean percentage of cell lysis for each set of triplicate wells using the formula: [(test release - spontaneous release)/(maximal release - spontaneous release)] x 100. This approach provided a quantitative measure of T cell-mediated cytotoxicity, allowing for a comprehensive analysis of T cell efficacy in lysing cancer cells across the range of E:T ratios tested.

### 2.14 Animal experiments

Female NOD/ShiLtJGpt-Prkdc^em26Cd52^ Il2rg^em26Cd22^/Gpt (NCG) mice, aged three to four weeks, were procured from GemPharmatech (Nanjing, China) for this study. All animal procedures were approved by the Institutional Animal Care and Use Committee of Sun Yat-sen University (Approval Number: AP20220244), and adhered to established ethical guidelines. Mice were housed under specific pathogen-free (SPF) conditions at 28°C and 50% relative humidity to ensure optimal welfare and experimental consistency. For the tumor suppression experiments, mice were randomly assigned to two groups, each consisting of three animals (n=5). Each mouse was subcutaneously injected with 2 x 10^6^ SAS cells on the dorsal surface. Tumor growth was monitored every five days for five weeks, with tumor volume calculated using the formula: TV = length x width^2^ x 0.5. Once tumors reached a volume of 50 mm^3^, mice were treated with one of the following regimens: 1) 2 x 10^7^ PD1^+^CXCL13^+^CD8^+^T cells; 2) 2 x 10^7^ PD1^+^CXCL13^-^CD8^+^T cells. Treatments were administered via tail vein injection once weekly for four weeks. Throughout the 35-day study period, tumor dimensions and weights were recorded at specified intervals. The maximal size of mice tumors did not exceed the limit set by the Institutional Animal Care and Use Committee of Sun Yat-sen University. At the end of the study, mice were euthanized, and tissue samples were collected to assess the therapeutic efficacy of the treatments.

### 2.15 Approval of ethics

Tumor tissue specimens were collected from patients with HNSCC who had not received preoperative chemotherapy, radiotherapy or immunotherapy. These specimens were obtained from the Department of Oral and Maxillofacial Surgery at Sun Yat-Sen Memorial Hospital, Sun Yat-Sen University (Approval Number: SYSKY-2023-684-01). Informed consent was secured from all subjects before their participation in the research.

### 2.16 Statistical analysis

Statistical analyses were performed using R software vision 4.1.1. Two-tailed Student’s t-test with the Bonferroni method was used for pairwise comparisons, while one-way ANOVA with Tukey’s method was employed for comparisons involving more than two groups. Kaplan-Meier survival curves were plotted, and the log-rank test was used for survival analysis. Cell culture experiments were conducted in triplicate for statistical reliability, with significance set at *P* < 0.05.

## 3 Results

### 3.1 The genetic and transcriptional characteristics of TLSRGs in HNSCC

A total of 23 TLSRGs were included in this study. Firstly, we explored the expression level of TLSRGs between tumor and normal tissues. There was a significant heterogeneity in the expression of TLSRGs (**Supplementary Figure S1A**). Among these 23 TLSRGs, CCL19, CCL21 and IL1R2 were highly expressed in normal tissues, while the rest genes were highly expressed in tumor tissues. In this study, the incidence of copy number variations (CNV) and somatic mutations of 23 TLSRGs for HNSCC were calculated and summarized (**Supplementary Figures S1B–D**). As a result, the 23 TLSRGs have prevalent CNV alterations in HNSCC. 12 TLSRGs have a frequency of CNV deletion, while the rest 11 genes were focused on the CNV amplification (**Supplementary Figure S1B**). Then, we showed the site of CNV alteration for TLSRGs on chromosomes (**Supplementary Figure S1C**). Moreover, we demonstrated that a total of 36 out of 506 patients with HNSCC carried the somatic mutation of the 23 TLSRGs (**Supplementary Figure S1D**). According to the results, the SH2D1A, CD40, TIGIT, IRF4, CXCL9, CD38 and STAT5A showed significant mutation, while the rest genes were not. Overall, the expression imbalance of TLSRGs was found in the HNSCC.

Then, we applied the Kaplan–Meier (KM) survival analysis to investigate the role of the expression or these 23 TLSRGs in prognosis of HNSCC. As shown in **Supplementary Figure S2**, all 23 TLSRGs were found to be associated with OS. Increased expression levels of CD200, CCR5, CCL21, CCL19, CCL18, CD38, ICOS, STAT5A, TIGIT, PDCD1, IRF4, IL10, IL2RA, IL1R2, SH2D1A, GFI1, FBLN7, CXCR3, CXCL13 and CXCL9 were indicative of favorable prognoses. Conversely, elevated expression of CD40, CSF2 and CCL20 referred to adverse survival. This result suggested that these23 TLSRGs could influence the prognosis of HNSCC.

### 3.2 Analysis of the TLSRGs in pan-cancer

To elucidate the potential impact of these TLSRGs, we assessed the expression differences and mutation frequencies of these 23 TLSRGs across 32 types of solid tumors. Our findings indicated that TLSRGs exhibited differential expressions in most types of cancers and are notably overexpressed in tumor tissues (**Supplementary Figure S3A**). We then conducted a comprehensive analysis of CNV variations and somatic mutations across all the 32 cancer types. As illustrated in **Supplementary Figure S3B**, TLSRGs such as TIGIT, STAT5A, SH2D1A, PDCD1, IRF4, IL2RA, IL1R2, IL10, ICOS, GFI1, FBLN7, CXCR3, CXCL9, CXCL13, CSF2, CD40, CD38, CD200, CCR5, CCL21, CCL20, CCL19 and CCL18 exhibited a higher frequency of CNV variations. While IRF4, STAT5A, FBLN7, IL1R2, CXCR3, TIGIT, CD38, PDCD1, CCR5, GFI1, CD40, IL2RA, CD200, SH2D1A, CXCL9 and ICOS showed noticeable single nucleotide mutations (**Supplementary Figure S3C**). Furthermore, we explored the relationship between somatic mutations and gene expression. This investigation revealed that somatic mutations in specific genes such as STAT5A, IRF4, IL1R2, IL10, CD40, CCR5, and CSF2 were significantly associated with changes in their respective gene expression levels especially in kidney chromophobe (KICH) (**Supplementary Figures S3D**). These findings enhance our understanding of TLSRGs’ role in solid tumors, particularly in how their mutations may influence gene expression and potentially impact tumor behaviors.

### 3.3 TLS patterns and characteristics in HNSCC

**Figure 1:**
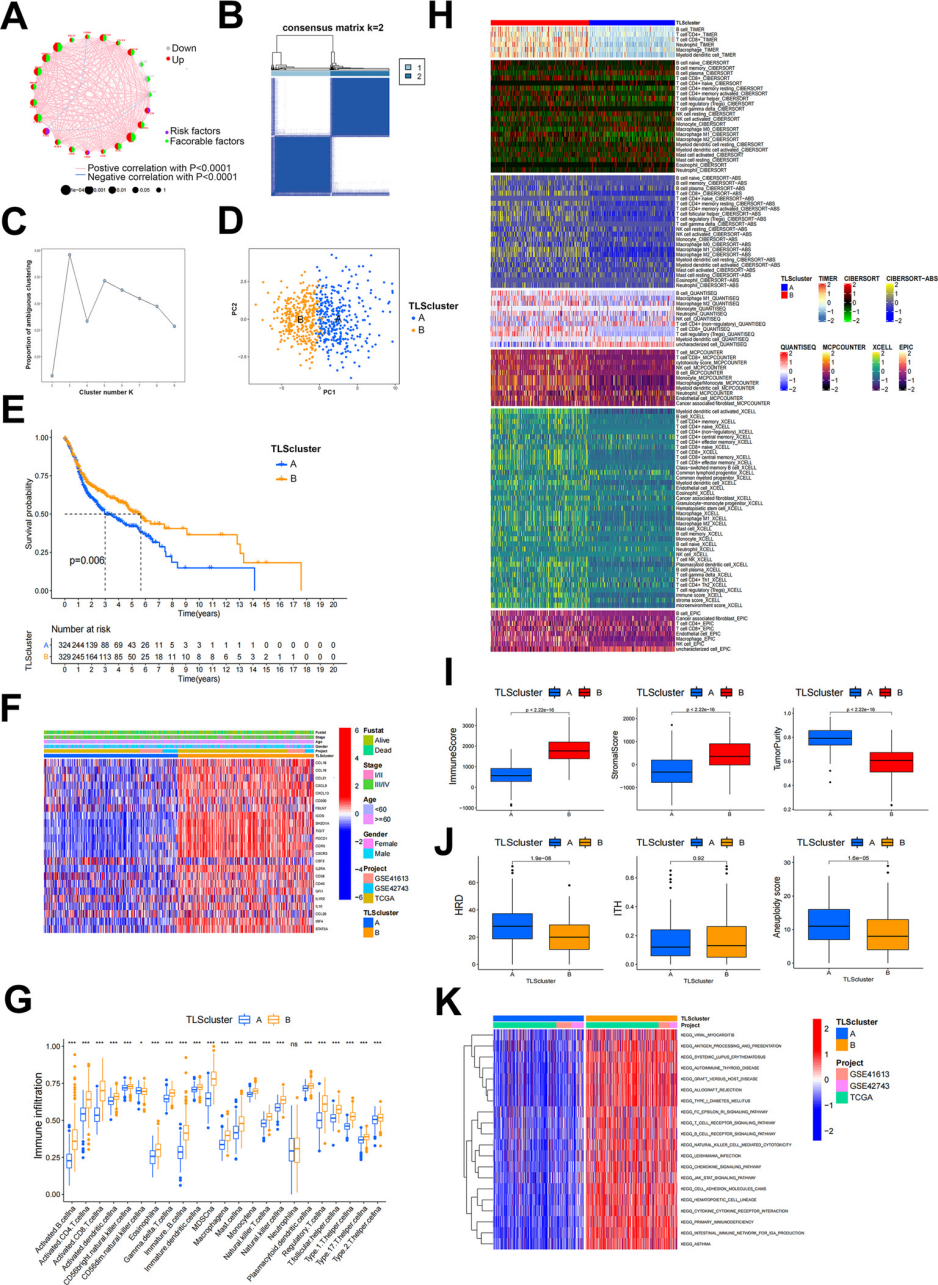
TLS patterns and their corresponding characteristics. **(A)** The correlation network of 23 TLSRGs. **(B)** Heatmap of DEGs in two TLSclusters identified by unsupervised clustering in consensus matrices for k = 2. **(C)** Scree plot of cluster numbers ranging from k=2 to 9. **(D)** PCA analysis of two TLSclusters. Blue dots, TLSclusterA; red dots, TLSclusterB. **(E)** KM survival curve for patients in two TLSclusters. **(F)** Heatmap of the expression of 23 TLSRGs between the TLScluster A and TLScluster B groups and their corresponded clinical information. **(G)** Immune cell infiltration analysis of two TLSclusters using ssGSEA method. **(H)** Heatmap of immune cells infiltration between two TLSclusters using TIMER, CIBERSORT, CIBERSORT-ABS, QUANTISEQ, MCPCOUNTER, XCELL and EPIC algorithms. **(I)** Comparison of ImmuneScore, StromalScore and tumor purity between the two TLSclusters using ESTIMATE algorithms. **(J)** Comparison of HRD, ITH and aneuploidy scores between the two TLSclusters **(K)** The GSVA analysis of the two TLSclusters. *P < 0.05, ***P < 0.001.

To comprehensively investigate the crosstalk and prognostic value among the 23 TLSRGs, we depicted their interactions in a network via univariate COX and correlation analyses (**Figure 1A**). Most TLSRG genes exhibited positive correlations with each other, with the exception of CSF2, which demonstrated a negative correlation with CD200 and CCL19. These results suggested that crosstalk among the TLSRGs may essentially impact tumor prognosis.

In this study, we incorporated three transcriptome datasets from HNSCC to analyze the different expression patterns of TLS. To ensure the accuracy of the results, we first combined the three transcriptome datasets and used the Combat package for batch correction. We then extracted the expression profiles of 23 TLSRGs based on the corrected transcriptome data and classified them using the ConsensusClusterPlus package. We selected K = 2 based on the proportion of ambiguous clustering plots. Consequently, 671 HNSCC patients were divided into two TLS patterns based on two clusters, TLSclusterA and B (**Figures 1B, C**). Before analysis, these two clusters were distinguished into separate categories using the PCA analysis method (**Figure 1D**). It was found that patients in TLSclusterB experienced longer survival, while those in TLSclusterA had a poorer prognosis (**Figure 1E**). Besides, significant differences in the expression of the 23 TLSRGs were observed between the two clusters (**Figure 1F**). We then identified the tumor microenvironments in each cluster. Compared to TLSclusterA, TME of TLSclusterB exhibited greater enrichment of immune cells, especially anti-tumor immune cells, as determined by various algorithms including TIMER, CIBERSORT, CIBERSORT-ABS, QUANTISEQ, MCPCOUNTER, XCELL, EPIC and ssGSEA (**Figures 1G, H**). These results implied that TLSclusterB represents an immune-activated microenvironment, in contrast to the potentially immuno-suppressive environment of TLSclusterA. In agreement with these findings, the immune and stromal scores were higher in TLSclusterB, whereas tumor purity was more prominent in TLSclusterA (**Figure 1I**). Meanwhile, we evaluated other immunogenic biomarkers such as homologous recombination deficiency (HRD), intratumor heterogeneity (ITH), and aneuploidy between these two clusters. Compared to TLSclusterB, TLSclusterA showed higher tumor immunogenicity (**Figure 1J**). We next implied GSVA analysis method to evaluate the hallmark gene set for the two clusters. According to **Figure 1K**, these two clusters revealed entirely different functions. Among them, TLSclusterB primarily enriched immune-related pathways, such as antigen processing and presentation, T cell receptor signaling pathway, B cell receptor signaling pathway and natural killer cell-mediated cytotoxicity, demonstrating its immune-activated properties.

### 3.4 Construction of the TLSscore

**Figure 2:**
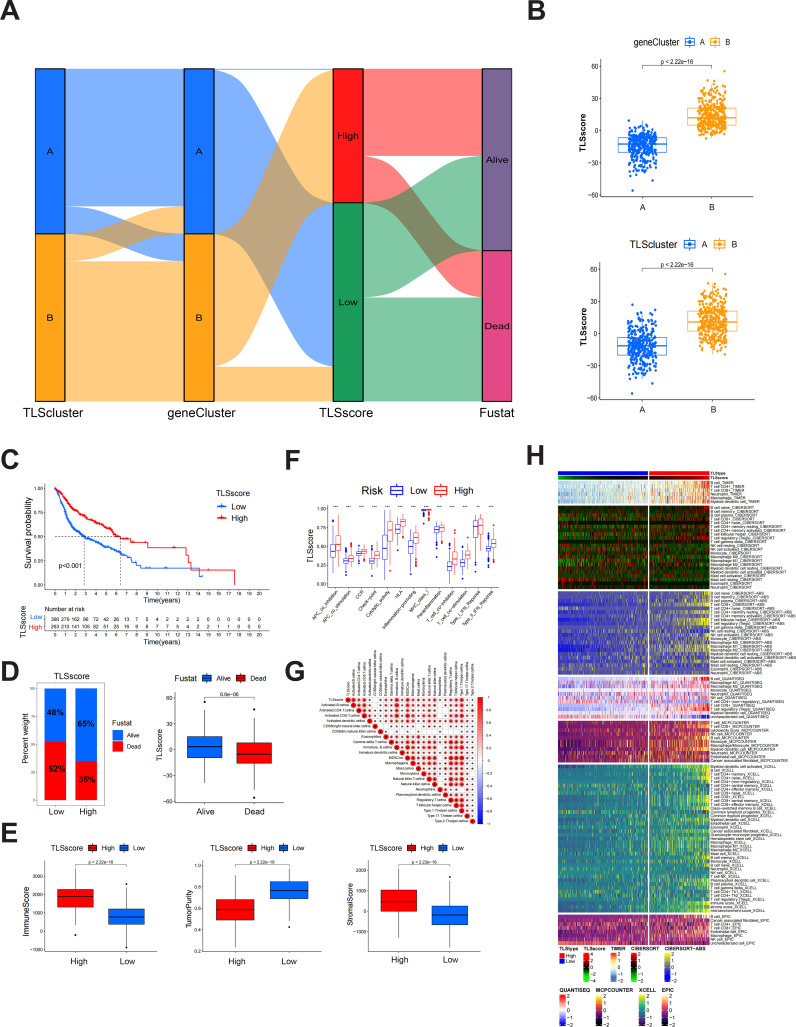
Construction of TLSscore and its corresponding characteristics. **(A)** Sankey chart illustrating the construction process of TLSscore. **(B)** The difference of TLSscore between TLSclusters and geneClusters. **(C)** KM survival curve of patients in TLSscore^high^ and TLSscore^low^ groups. **(D)** The difference of survival status between TLSscore^high^ and TLSscore^low^ groups. **(E)** Comparison of ImmuneScore, StromalScore and tumor purity between TLSscore^high^ and TLSscore^low^ groups using ESTIMATE algorithms. **(F)** Immune function analysis between TLSscore^high^ and TLSscore^low^ groups using ssGSEA method. **(G)** Correlation analysis between TLSscore and immune cell infiltration. **(H)** Heatmap of immune cell infiltration between TLSscore^high^ and TLSscore^low^ groups using TIMER, CIBERSORT, CIBERSORT-ABS, QUANTISEQ, MCPCOUNTER, XCELL and EPIC algorithms. ^*^ *P* < 0.05, ^**^ *P* < 0.01, ^***^ *P* < 0.001.

The above results demonstrate that patients of different TLSclusters exhibit distinct characteristics and prognosis. However, we are still unable to evaluate the features of each patient due to heterogeneity. Therefore, we constructed a novel TLSscore model to assess individual patterns. We first conducted differential analysis between TLSclusterA and TLSclusterB to identify differentially expressed genes (DEGs). Utilizing these DEGs, we conducted 1000 unsupervised cluster analyses and ultimately divided the patients with HNSCC into two molecular subtypes based on TLSRGs phenotypeyedlysdveus, A and B (**Supplementary Figures S4A, B**). Kaplan-Meier survival analysis revealed that patients in geneCluster B exhibited longer survival times, whereas those in geneCluster A faced poorer prognoses (**Supplementary Figure S4C**). In terms of gene expression, numerous DEGs were observed between the two geneClusters (**Supplementary Figure S4D**). Additionally, geneCluster B was characterized by a higher infiltration of immune cells, elevated immune and stromal scores, lower tumor purity, reduced homologous recombination deficiency (HRD), and lower aneuploidy (**Supplementary Figures S4E–G**). Subsequently, we performed gene expression differential analysis between geneCluster A and B, and conducted unsupervised clustering method and univariate COX analysis to identify prognostic genes. Based on these different prognostic genes, we constructed the TLS signature modeltureedd,ys using the PCA method (**Figure 2A**). According to this TLSscore method, we found that patients of TLScluster B and geneClusterB also exhibited higher TLSscore compared to their respective corresponding clusters (**Figure 2B**). Based on optimal threshold, we observed that patients in the TLSscore^high^ group had a better prognosis, indicating the potential of TLSscore as a prognostic indicator for HNSCC, which also suggests that TLS may affect the prognosis of HNSCC (**Figures 2C, D**). In order to explore whether the TLSscore can be used as an independent prognostic factor of HNSCC, we conducted univariate and multivariate Cox regression analysis to evaluate the TLSscore with multiple clinical parameters (**Supplementary Figures S5A, B**). Both univariate and multivariate analysis results showed that TLSscore (HR = 0.528, 95% CI = 0.412-0.677, *P* < 0.001; HR = 0.529, 95% CI =0.412-0.678, *P* < 0.001) were significantly correlated with better prognosis of HNSCC patients. Moreover, subgroup analysis further corroborated these findings, demonstrating consistency in the prognostic value of the TLSscore across different patient clinical features such as different ages, gender and clinical stages (**Supplementary Figures S5C–E**). We next investigated the relationship between TLSscore and immune patterns. As shown in **Figure 2E**, patients with TLSscores^high^ had elevated immune and stromal scores, but lower tumor purity. Consistently, we demonstrated that patients with TLSscore^high^ also possessed more prominent immune-related functions by using the ssGSEA method (**Figure 2F**). Interestingly, we found a positive correlation between TLSscore and immune infiltrating cells. Specifically, TLSscore^high^ exhibited a strong positive correlation with activated B cells, activated CD4 cells, activated CD8 cells, and immature B cells (**Figures 2G, H**). These findings suggest that an increased TLSscore might contribute to a more robust anti-tumor immunity.

### 3.5 Multiomics analysis of the role of TLSscore

To delve deeper into the differences between the HNSCC patient with high and low TLSscore, we further employed the Monocle package to distinctly separate HNSCC samples based on varying TLSscores (**Supplementary Figure S6A**). When comparing single nucleotide polymorphisms (SNP) between the high and low TLSscore groups, we observed that patients in the TLSscore^high^ group had a higher tumor mutation rate (91.24%) compared to those in the TLSscore^low^ group (84.52%) (**Supplementary Figure S6B**). Next, we examined the relationship between TLSscore and Tumor Mutational Burden (TMB). As shown in **Supplementary Figure S6C**, it was evident that the TMB scores were significantly higher in the TLSscore^low^ group than in the TLSscore^high^ group. Furthermore, we identified a negative correlation between TLSscore and TMB (R=-0.19, P<0.05) (**Supplementary Figure S6D**). Consistent with our previous study, patients with high TMB were found to have poorer prognoses (**Supplementary Figure S6E**). Interestingly, we found that by combining TLSscore with TMB, we can differentiate two patient groups into distinct outcomes: TLSscore^high^ patients with a low level of TMB had the longest survival rate, while TLSscore^low^ patients with a high level of TMB exhibited the worst prognosis (**Supplementary Figure S6F**). These findings provide a promising method to predict patient survival by integrating the TLSscore with TMB. Subsequently, we explored CNV between the two TLSscore groups. Both TLSscore^high^ and TLSscore^low^ group exhibited focal amplifications and deletions in various chromosomal regions (**Supplementary Figure S6G**). However, the TLSscore^low^ group exhibited higher focal-level gain and loss burdens, as well as a higher arm-level gain burden, compared to the TLSscore^high^ group (**Supplementary Figure S6H**). The distribution of G-scores (based on the frequency and amplitude of gains and losses) across all chromosomes in both TLSscore^high^ and TLSscore^low^ groups were presented in **Supplementary Figure S6I**. These findings indicate that the TLSscore^low^ group has relatively high immunogenicity, whereas the TLSscore^high^ group exhibits low immunogenicity.

**Figure 3:**
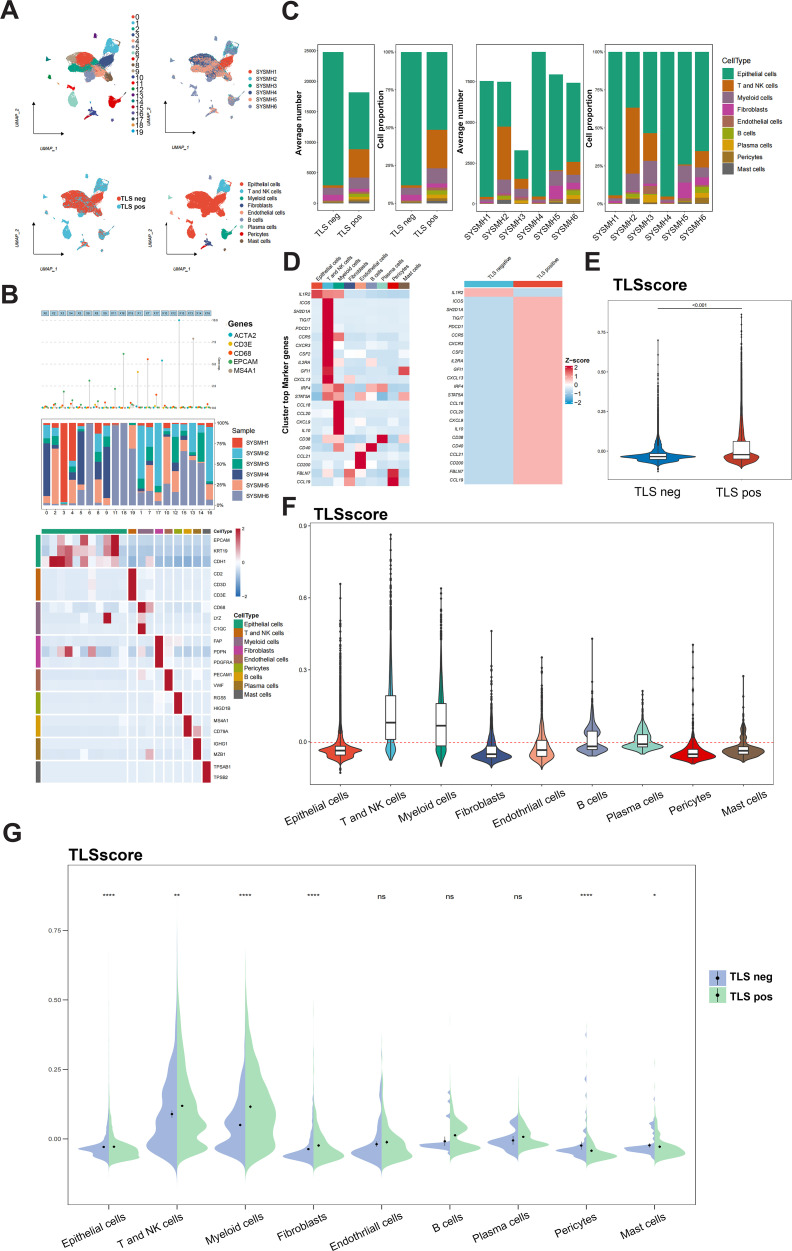
Analysis of TLSscore in HNSCC with TLS at single-cell atlas. **(A)** The distribution of 20 cell clusters, 6 tumor samples, 9 cell types, and patients of TLS-negative/positive were visualized and labeled using the UMAP method. **(B)** Annotation of different cell types. **(C)** The proportion and average number of 9 main cell types between 6 tumor samples and patients of TLS-negative/positive. **(D)** Heatmap displaying the expression of the 23 TLSRGs in 9 main cell types (left) and in TLS-negative/positive groups (right). **(E)** Comparison of TLSscore between TLS-negative/positive groups. **(F)** Comparison of TLSscore across 9 main cell types. **(G)** Comparison of TLSscore across 9 main cell types between TLS-positive and TLS-negative groups. *P < 0.05, **P < 0.01, ****P< 0.0001

We further explored the role of the TLSRGs and TLSscore in HNSCC by conducting a reanalysis of our previously published single-cell transcriptomic sequencing data (GSE172577). This dataset comprises single-cell RNA sequencing (scRNA-seq) data from six patients. Among them, three patients were confirmed to have TLS through immunohistochemical analysis and were classified as TLS-positive. The remaining three patients did not exhibit TLS and were categorized as TLS-negative. After quality control, a total of 42,979 cells were available for analysis (**Figure 3A**). Utilizing canonical marker gene expression, we identified 9 main cell types (**Figure 3B**). As illustrated in **Figure 3C**, the TLS-positive group exhibited a higher percentage of T and NK cells, myeloid cells, B cells, plasma cells, endothelial cells, pericytes and mast cells compared to the TLS-negative group. In particular, we found that the 23 TLSRGs were mainly expressed in TLS-positive group, especially in T and NK cells, myeloid cells, B cells, endothelial cells and plasma cells, which are main cell types composing of TLS, as prior research reported (29) (**Figure 3D**). These findings underscore the pivotal role of TLSRGs in shaping the tumor immune microenvironment, particularly in modulating immune responses associated with TLS. As the TLSscore model was constructed based on the expression of these 23 TLSRGs, it was evident that the TLS-positive group demonstrated higher scores than the TLS-negative group (**Figure 3E**). Besides, we found that T and NK cells showed the highest TLSscores among the nine cell types, and these cells in the TLS-positive group also exhibited elevated TLSscores compared to their TLS-negative counterparts (**Figures 3F, G**). Moreover, two spatial transcriptomics sequencing data were applied to explore the spatial relationship between TLSscore and TLSs (**Supplementary Figures S7A, B**). TLS positive samples exhibited a higher TLScore compared to TLS negative ones, with a significant enrichment of TLSscore observed specifically within the TLS regions. These findings highlight the strong association between TLSscore and the presence of tertiary lymphoid structures (**Supplementary Figure S7C**).

### 3.6 Predictive potential of TLSscore for immunotherapy efficacy

**Figure 4:**
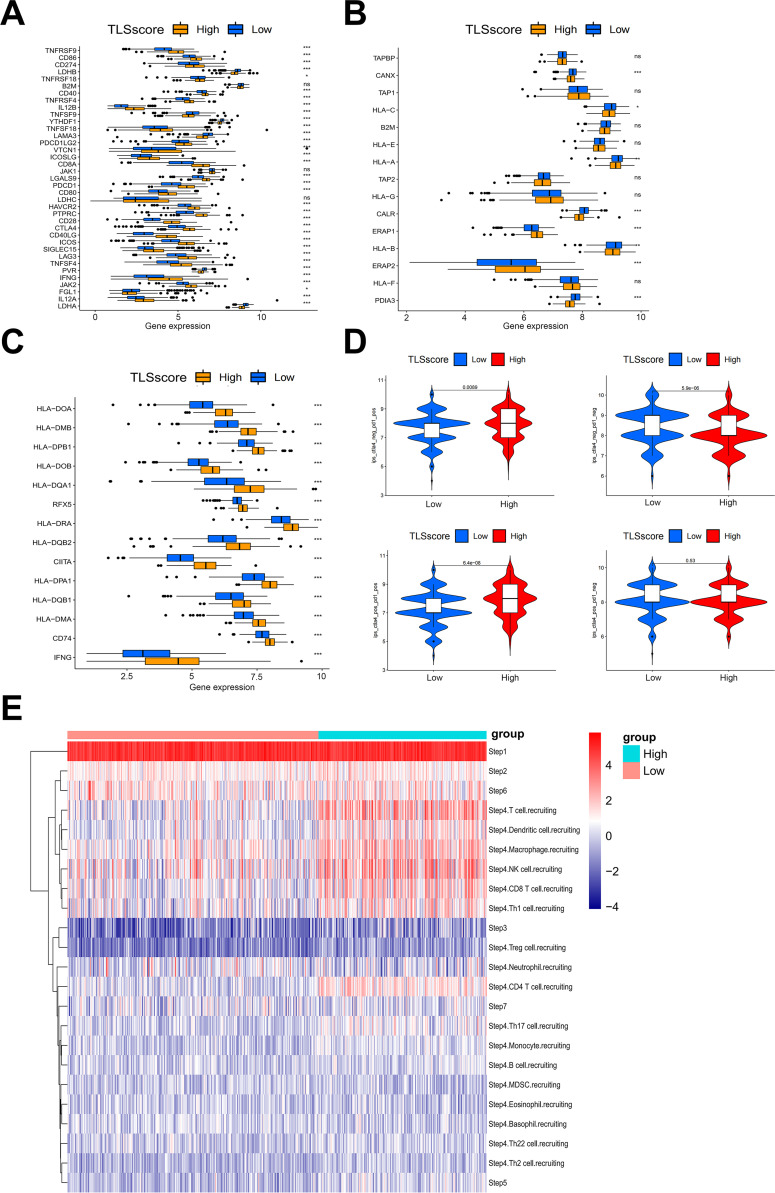
The association between TLSscore and immune-related functions. **(A)** Comparison of immune checkpoint genes between TLSscore^high^ and TLSscore^low^ groups. **(B)** Comparison of MHC I genes between TLSscore^high^ and TLSscore^low^ groups. **(C)** Comparison of MHC II genes between TLSscore^high^ and TLSscore^low^ groups. **(D)** Comparison of IPS scores between TLSscore^high^ and TLSscore^low^ groups among CTLA4_Positive PD1_Positive, CTLA4_Negative PD1_Negative, CTLA4_Negative PD1_Positive and CTLA4_Positive and PD1_Negative subgroups. **(E)** TIP analysis of immune cell infiltration across each step of the cancer-immunity cycle between TLSscore^high^ and TLSscore^low^ groups. ^*^ *P* < 0.05, ^**^ *P* < 0.01, ^***^ *P* < 0.001.

**Figure 5:**
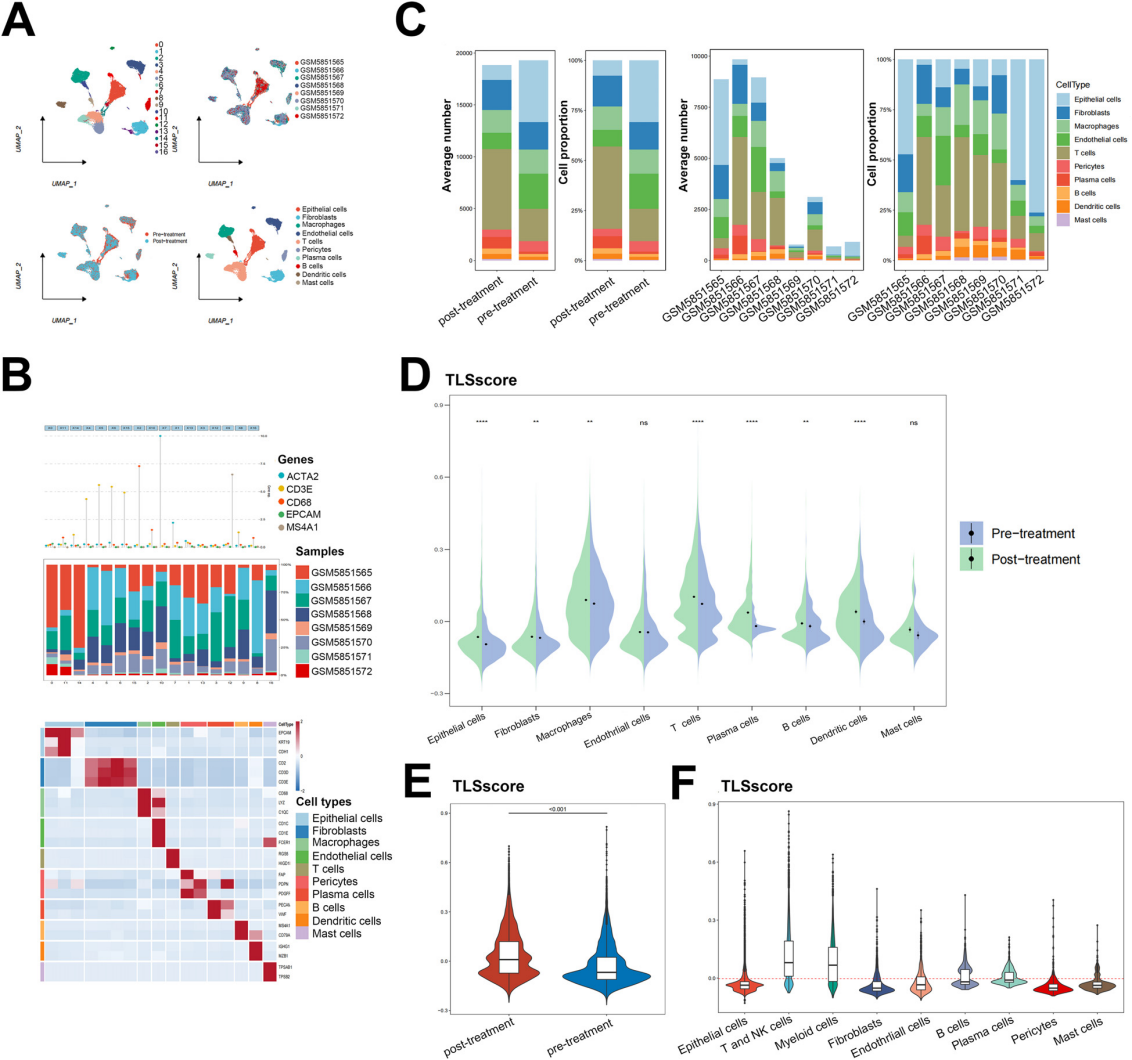
Single cell RNA sequencing analysis for the role of TLSscore in immunotherapy. **(A)** The UMAP visualization shows the distribution of 17 cell clusters, 8 tumor samples, 10 cell types and patients before/after immunotherapy in the GSE195832 dataset. **(B)** The cell type annotation in GSE195832. **(C)** The proportion and average number of 10 main cell types between patients before/after immunotherapy. **(D)** Comparison of TLSscore across 9 main cell types between pre- and post-treatment groups in GSE195832. **(E)** Comparison of TLSscore between pre- and post-treatment groups in GSE195832. **(F)** Comparison of TLSscore across 9 main cell types in GSE195832. **P < 0.01, ****P < 0.0001.

Our analysis on immune cell infiltration above unveiled a positive correlation between TLSscore and various immune infiltrating cells, suggesting that TLS may play a pivotal role in mediating anti-tumor immunity in HNSCC, which is consistent with conclusions drawn from other previous studies (16). Thus, we determined whether the TLSscore is associated with immune checkpoints and can serve as an effective predictor for evaluating the response to immunotherapy. According to **Figures 4A–C**, TLSscore^high^ group had higher expression of immune checkpoint-related genes, MHC I and II related genes than the TLSscore^low^ group. Thus, we utilized immune phenotype score (IPS) data to evaluate the four patient subgroups treated with different immune checkpoint inhibitors (ICIs), including anti-PD-1 and anti-CTLA-4. As shown in **Figure 4D**, patients in the TLSscore^high^ group exhibited higher IPS scores in both CTLA4_Positive PD1_Positive, CTLA4_Negative PD1_Negative and CTLA4_Negative PD1_Positive subgroups (P<0.05). These results suggest that the TLSscore could serve as a valuable indicator of immunotherapy efficacy in specific patient subsets, potentially facilitating its clinical application in HNSCC treatment. To visualize the activity of anti-cancer immunity, we used the TIP method to analyze the tumor-infiltrating immune cells across the seven-step cancer-immunity cycle. Compared to TLSscore^low^ group, TLSscore^high^ group were active in trafficking of immune cells to tumors (Step 4) (**Figure 4E**). Moreover, two single cell RNA sequencing data (GSE195832 and GSE123813) were applied to explore the role of TLSscore during the immune response in this study. In HNSCC cohort GSE195832, we obtained 38122 cells for further analysis (**Figure 5A**). Based on canonical marker genes, 10 main cell types were identified (**Figure 5B**). After treatment with PD-1 inhibitordgnityingls the percentage of epithelial cells was decreased, while the T cells, B cells and plasma cells were increased. (**Figures 5C, D**). Similarly, The TLSscore was elevated in the post-treatment group after the treatment with immune checkpoint blockade (ICB) (**Figure 5E**). These findings are consistent with previous reports indicating that immunotherapy can induce alterations in the immune microenvironment, thereby leading to the promotion of TLS formation. Notably, our TLSscore model has the capability to evaluate the dynamic changes in TLS for individuals. In particular, T and NK cells were observed exhibiting the highest TLSscore among all cell types (**Figure 5F**). To assess whether TLSscore can predict the response to immunotherapy, we integrated another cohort GSE123813 to determine whether TLSscore would differentiate between responder and non-responder groups before or after ICB treatment (**Supplementary Figure S8A**). we compared TLSscores between responder and non-responder groups. As shown in **Supplementary Figures S8B–D**, responding patients exhibited higher TLSscores than non-responders. Furthermore, our analysis revealed that TLSscore was higher in the responder group both before and after ICB treatment. These results collectively indicate that TLSscore exhibits outstanding predictive efficacy for immunotherapy.

### 3.7 PD-1^+^CXCL13^+^CD8^+^T cells are pivotal in both TLS and immunotherapy

**Figure 6:**
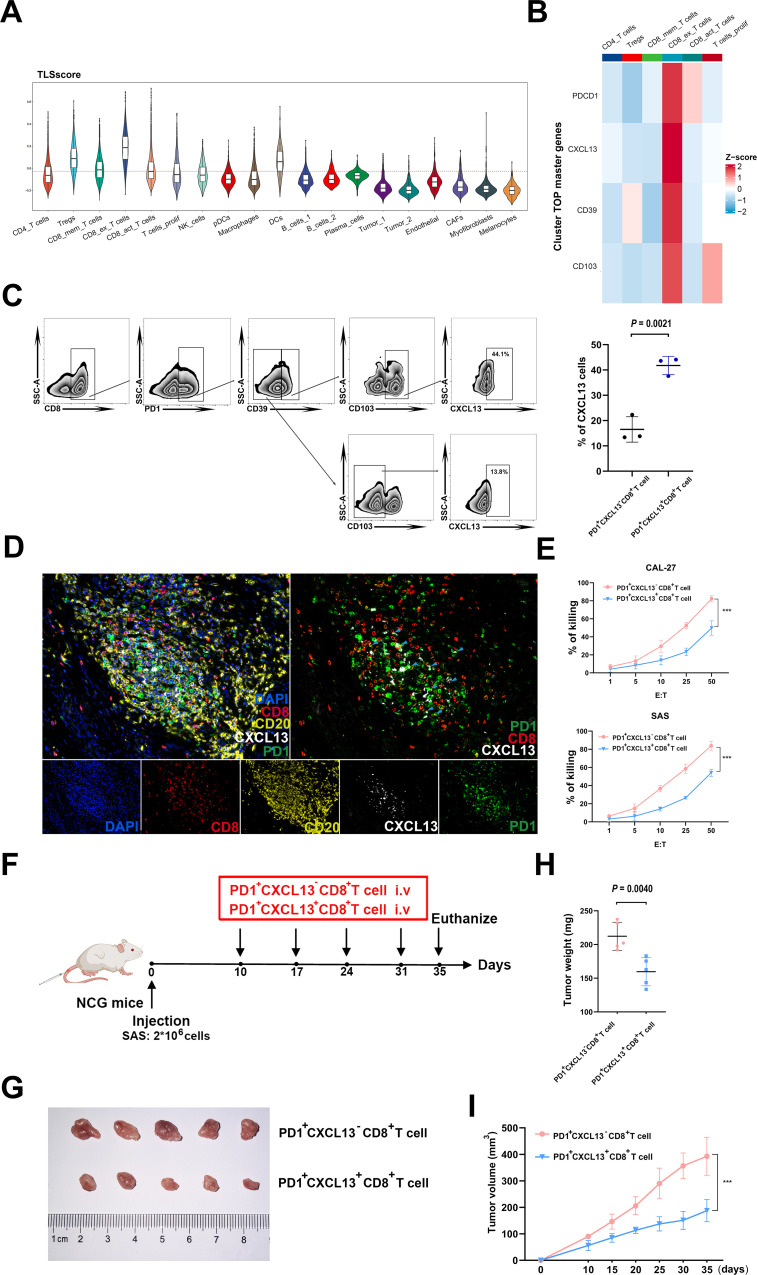
Functional properties of PD1+CXCL13+CD8+T cells in vitro and vivo. **(A)** Comparison of TLSscore across 19 main cell types in GSE195832. **(B)** Heatmap depicting the top cell-type-specific markers of T cells in GSE195832 dataset. **(C)** FACS analysis of PD1+CD39+CD103+CD8+T cells and PD1+CD39-CD103-CD8+T cells. Right panel, statistical analysis of FACS results of PD1+CD39+CD103+CD8+T cells and PD1+CD39+CD103+CD8+T cells (n=3). **(D)** MIHC staining of PD1+CXCL13+CD8+T cell marker PD1 (green), CXCL13 (white) and CD8 (red), B cells marker CD20 (yellow) and DAPI staining (blue) in TLS region of HNSCC sample. Scale bars, 100mm. **(E)** LDH assay of PD1+CXCL13+CD8+T cells and PD1+CXCL13-CD8+T cells cocultured with HNSCC cells at the indicated E:T ratios. Horizontal lines indicate the mean ± SEM. Significance was determined with a 2-tailed Mann-Whitney U test. **(F)** Schematic illustration of tumor inoculation and different treatments in SAS tumor-bearing NCG mice. At day 10 after tumor inoculation, mice were treated with PD1+CD39+CD103+CD8+T cells and PD1+CD39-CD103-CD8+T cells. **(G–I)** Tumor images **(G)**, tumor weights **(H)**, and tumor growth curves **(I)** of SAS xenograft-bearing mice after intravenous injection of PD1+CXCL13+CD8+T cells and PD1+CXCL13-CD8+T cells (5 mice in each group). ***P < 0.001.

Considering the crucial role of TLSscore in predicting immunotherapy response, we subsequently conducted a detailed analysis to identify key cell types involved in this process. By integrating two cohorts of single cell RNA sequencing data, we observed that exhausted CD8^+^T cells exhibited the highest TLSscore. Both before and after ICB treatment, the responder group showed higher TLSscore in exhausted CD8^+^T cells. Furthermore, an elevation in TLSscore of exhausted CD8^+^T cells was observed after ICB treatment (**Figure 6A**; **Supplementary Figures S8B–D**). To further explore the anti-tumor ability of exhausted CD8^+^T cells within TLS, we analyzed the top master genes of this cell subtype. As shown in **Figure 6B**, exhaustion marker PD1 and chemokines CXCL13 were the highest expression gene of this exhausted CD8^+^T cell subtype. In order to isolate PD1^+^CXCL13^+^CD8^+^T cells, we investigated whether the surface markers CD39 and CD103, which are highly expressed, could serve as substitutes for CXCL13. According to our prior investigations, CD103^+^CD8^+^T cells exhibited a greater abundance of CXCL13 compared to CD103^-^CD8^+^T cells (30). In this study, flow cytometry analysis revealed that PD1^+^CD39^+^CD103^+^CD8^+^T cells had a higher proportion of CXCL13 compared to PD1^+^CD39^-^CD103^-^CD8^+^T cells (**Figure 6C**). Based on these findings, we opted to use CD39 and CD103 for flow cytometry sorting method to isolate PD1^+^CXCL13^+^CD8^+^T cells. Subsequently, we employed mIHC method to determine the spatial co-localization of the PD1^+^CXCL13^+^CD8^+^T cells within TLS. As illustrated in **Figure 6D**, our findings indicated that PD1^+^CXCL13^+^CD8^+^T cells were dispersed throughout the TLS. These results highlight PD1^+^CXCL13^+^CD8^+^T cells as the subtype with the highest TLSscore within TLS. Additionally, we identified CD39 and CD103 as effective surface markers for isolating these subtype cells.

To further investigate the anti-tumor potential of the PD1^+^CXCL13^+^CD8^+^T cell subtype, we conducted *in vitro* cytotoxicity assays using PD1^+^CXCL13^+^CD8^+^T cells and PD1^+^CXCL13^-^CD8^+^T cells co-cultured with HNSCC cells. Our results revealed that PD1^+^CXCL13^+^CD8^+^T cells exhibited enhanced E:T ratios, indicating a stronger anti-tumor capacity against HNSCC cells (**Figure 6E**). To further explore the function of human PD1^+^CXCL13^+^CD8^+^T cells in antitumor immunity *in vivo*, we established HNSCC subcutaneous tumor model by subcutaneously injected HNSCC cell line SAS into NCG mice (**Figure 6F**). Then, we intravenously injected PD1^+^CXCL13^+^CD8^+^T cells and PD1^+^CXCL13^-^CD8^+^T cells separately. Our data demonstrate that treatment with PD1^+^CXCL13^+^CD8^+^T cells effectively reduces the growth of HNSCC tumors compared to PD1^+^CXCL13^-^CD8^+^T cells (**Figures 6G–I**). These results suggest that PD1^+^CXCL13^+^CD8^+^T cells may represent one of the tumor-reactive CD8^+^T cell subtypes within TLS, which can be quantified individually using our TLSscore model.

## 4 Discussion

Diverging from the traditional theory that adaptive immune responses to tumors predominantly occur in secondary lymphoid organs (SLO), TLS is a lymphoid-like structure that plays a pivotal role in chronic inflammation, the development of autoimmune diseases and the anti-tumor immune process within non-lymphoid tissues (18, 31, 32). Recent research has extensively explored the predictive value of TLS for the survival prognosis and immunotherapy response across various cancers (24–26). However, limited biomarkers and variations in TLS detection methods led to contradicted conclusions. Additionally, the mechanism by which TLS exerts anti-tumor effects remains controversial. In our previous study, we identified that a specific subgroup of TCF7^+^T cells possess the ability to recruit and home T cells, which was associated with favorable outcome of HNSCC patients (23). In this study, we constructed a TLSscore model based on transcriptome sequencing and demonstrated its predictive function for survival prognosis and immunotherapy response in HNSCC. Furthermore, a distinct subgroup of tumor-infiltrating lymphocytes (TILs), PD1^+^CXCL13^+^CD8^+^T cells was found to localize with TLS and exhibited enhanced anti-tumor effects. Overall, these findings provide a new perspective on the application of TLS in predicting prognosis and response of immunotherapy in HNSCC.

In this study, we selected 23 previously validated TLSRGs and analyzed their gene mutations and prognosis in HNSCC. Subsequently, we integrated three HNSCC cohorts from three transcriptome datasets into a new meta-cohort and categorized the patients into two TLS clusters, A and B. Patients in TLSclusterB exhibited longer survival and showed immune-activated properties compared to those in TLSclusterA. Through differential gene analysis, we identified numerous DEGs. By using the DEGs from TLSclusterA and B, we employed an unsupervised clustering method to further divide the patients into two new gene clusters. Similarly, we found that patients in gene clusterB had better survival advantages and showed greater immune cell infiltration. In order to calculate individuals’ TLS-related scores, we constructed the TLS signature score model (TLSscore) based on the DEGs from gene clusters A and B. Patients with a high TLSscore had a longer survival rate and a richer population of activated immune cells in each step of the cancer-immunity cycle. Additionally, we observed that a high TLSscore was correlated with a lower TMB. Consistent with our previous findings, HNSCC patients with low TMB had better prognoses than those with high TMB (33). Importantly, in our study, patients with a higher TLSscore and lower TMB exhibited the longest survival, suggesting that the combination of TLSscore and TMB could serve as a better prognostic indicator for HNSCC. Furthermore, we found that TLSscore was spatially associated with TLS, enabling the quantification of the TLS pattern in individuals.

Although immunotherapy, such as immune checkpoint blockade (ICB), has significantly improved the survival rate, only a small proportion of HNSCC patients can benefit from it. To date, the combined positive score (CPS), a method to evaluate the expression of PD-L1, is the most commonly used method to predict the response to ICIs treatment in clinical practice (34–38). However, HNSCC exhibits a high degree of tumor heterogeneity, which can lead to inaccurate determination of PD-L1 during biopsy, thereby reducing the predictive efficacy of ICIs treatment (39). Recently, numerous studies have confirmed that TLS is associated with improved prognosis and elevated response rates to ICIs in various types of cancers (18–22, 24–26). According to previous studies, TLS provides a special niche to foster cell-cell contact by antigen-laden APCs and naïve lymphocytes in the tumor area (18). Meanwhile, the B cells within TLS can produce tumor-specific antibodies mediating complement lysis and antibody-dependent cytotoxicity (40, 41). Additionally, naïve T cells can be recruited, re-educated, and proliferated in TLS (42). Interestingly, *de novo* TLS formation in various cancers can be stimulated by several therapeutic approaches, including neoadjuvant chemotherapy, cancer vaccines, and immune checkpoint blockade (ICB) therapy (43–45). Inspired by these findings, we constructed a TLSscore model to predict immunotherapy response of individuals and guide clinical treatment strategy of HNSCC. Using two single-cell RNA sequencing cohorts of HNSCC and BCC, we confirmed that patients responding to ICIs treatment possess a higher TLSscore. Interestingly, we also discovered that TLSscore increased after immunotherapy, which was consistent with the study that TLS can be therapeutically induced by ICIs treatments (46, 47). Collectively, our results provide a more precise and effective model to predict immunotherapy response in HNSCC.

Accumulating evidence indicates that TILs play a pivotal role in recognizing and eliminating tumors. Among them, CD8^+^T cells emerge as the most crucial subpopulation of immune cells (48). However, the predictive value of the quantity of CD8^+^T cells remains controversial. Recent evidence indicates that the majority of CD8^+^T cell are “bystander” T cells that lack the ability to recognize the specific antigen of tumors due to their heterogeneity (49, 50). Despite the fact that TLS provides a spatial contact for T cell recognition of tumor antigens, the subset of tumor-specific T cells within TLS remains unclear. In our study, we identified a specific exhausted subgroup of CD8^+^T cells expressing PD-1 and secreting the chemokine CXCL13, exhibiting the highest TLSscore. Previous studies have shown that CXCL13 is a CXC chemokine capable of inducing the migration of CXCR5^+^ immune cells (51). Additionally, reports have demonstrated that elevated level of CXCL13 in TLS plays a significant role in recruiting B cells, T cells and dendritic cells, thereby promoting the formation of TLS (52, 53). In a lung cancer research, Zhou et al. demonstrated that CXCL13 serves as the unique marker for antigen-specific T (Tas) cells and its high expression indicates a high response rate to ICB (54). In the study of metastatic colorectal cancer and endometrial cancer, CXCL13^+^CD8^+^T cells in tumor microenvironment (TME) have been shown to exhibit high proliferation ability, tumor-activating characterization and expression anti-tumor molecular capability, which can be a predictor of better prognosis (55, 56). Despite CXCL13^+^CD8^+^T cells are terminally differentiated cells, they can still exhibit remarkable clonal expansion ability (55). Consistent with our results, we demonstrated that PD1^+^CXCL13^+^CD8^+^T cells possess superior anti-tumor abilities both *in vivo* and *in vitro*. Overall, we have identified a specific CD8+T cell subgroup demonstrating a high level of anti-tumor ability within TLS, which unveiled the underlying mechanisms of TLS-mediated tumor-killing immunity and possessed promising clinical implications such as adoptive cell therapy (ACT).

Despite the construction of a TLSscore model and the identification of a tumor-specific T cell subgroup associated with TLS, this study has several limitations. First, our TLSscore model was developed by integrating 3 HNSCC cohorts from the TCGA and GEO databases. However, we did not use an independent validation cohort to confirm its prognostic value. Additionally, although the TLSscore model demonstrated strong predictive potential, we did not compare its predictive capability with other models reported in the literature. To further evaluate its clinical utility, prospective clinical trials involving HNSCC cohorts are necessary to validate both its prognostic significance and its ability to predict immunotherapy response in HNSCC patients. Therefore, validating the TLSscore model in independent prospective HNSCC cohorts and comparing its predictive efficacy to other models are essential to fully realizing its clinical potential in future studies.

## 5 Conclusion

In summary, our study demonstrated the characteristics of TLSRGs in HNSCC. Additionally, we developed a TLSscore model to evaluate TLS patterns for individuals, allowing to assess the survival prognosis, degree of immune cell infiltration and response to immunotherapy. Furthermore, we identified a tumor-specific PD^-^1^+^CXCL13^+^CD8^+^T cell subgroup within TLS and elucidated its anti-tumor functions. Our study will provide a deeper understanding of TLS and offer clinical strategies to guide personalized precision medicine such as ICB and ACT.

## Data Availability

The original contributions presented in the study are included in the article/**Supplementary Material**. Further inquiries can be directed to the corresponding authors.
