# Supplementary material for: Tertiary lymphoid structure-related score as a predictor for survival prognosis and immunotherapy response in head and neck squamous cell carcinoma
Source: Front Immunol. 2024 Oct 18;15:1483497. doi: 10.3389/fimmu.2024.1483497 (PMC11527632; doi:10.3389/fimmu.2024.1483497)
Supplement: Supplementary file 1 [file DataSheet1.docx]

Supplementary Material

**Supplementary Figures**  **
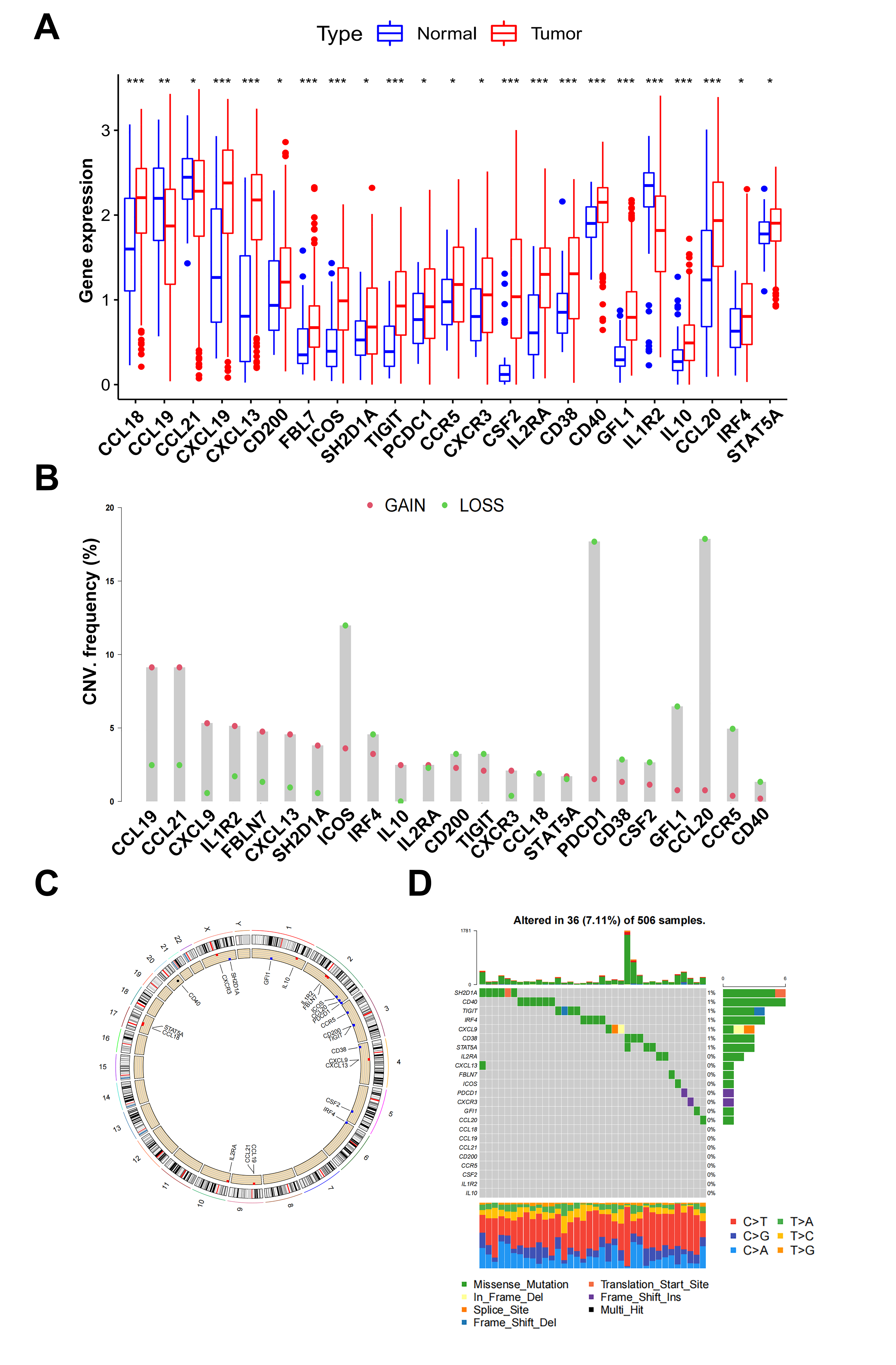
**

**Figure S1. The basic characteristics of expression and genetic variation of TLSRGs in HNSCC.** (A) The differential expression of the 23 TLSRGs between tumor and normal tissues. (B) The CNV variation frequency of the 23 TLSRGs. GAIN, amplification frequency; LOSS, deletion frequency. (C) The location of the 23 TLSRGs in 23 pairs of chromosomes. (D) The somatic mutation frequency of the 23 TLSRGs in HNSCC. ^*^*P* < 0.05，^**^*P* < 0.01，^***^*P* < 0.001.

**
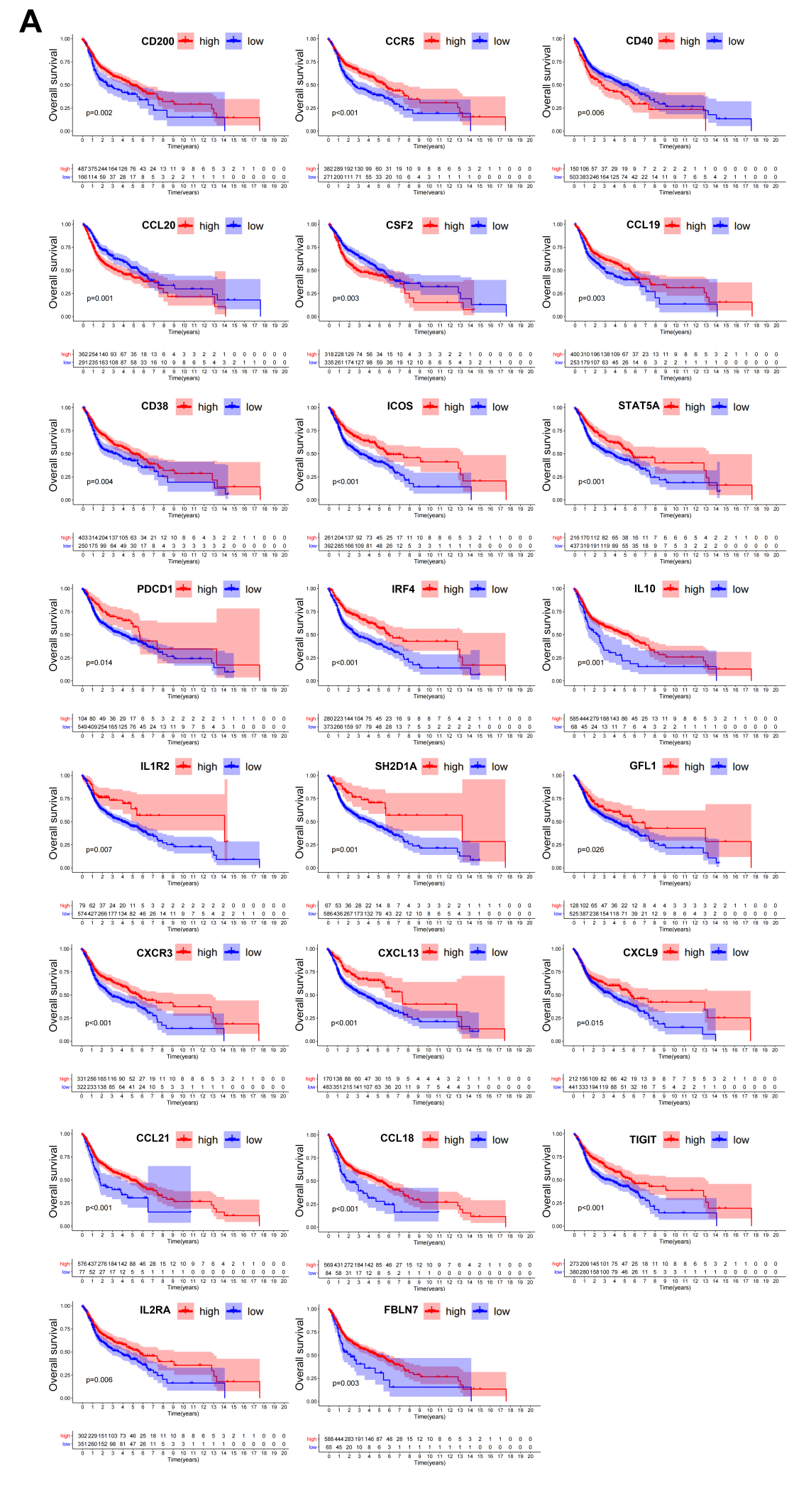
**

**Figure S2. Kaplan–Meier (KM) survival curves of 23 TLSRGs.**

**
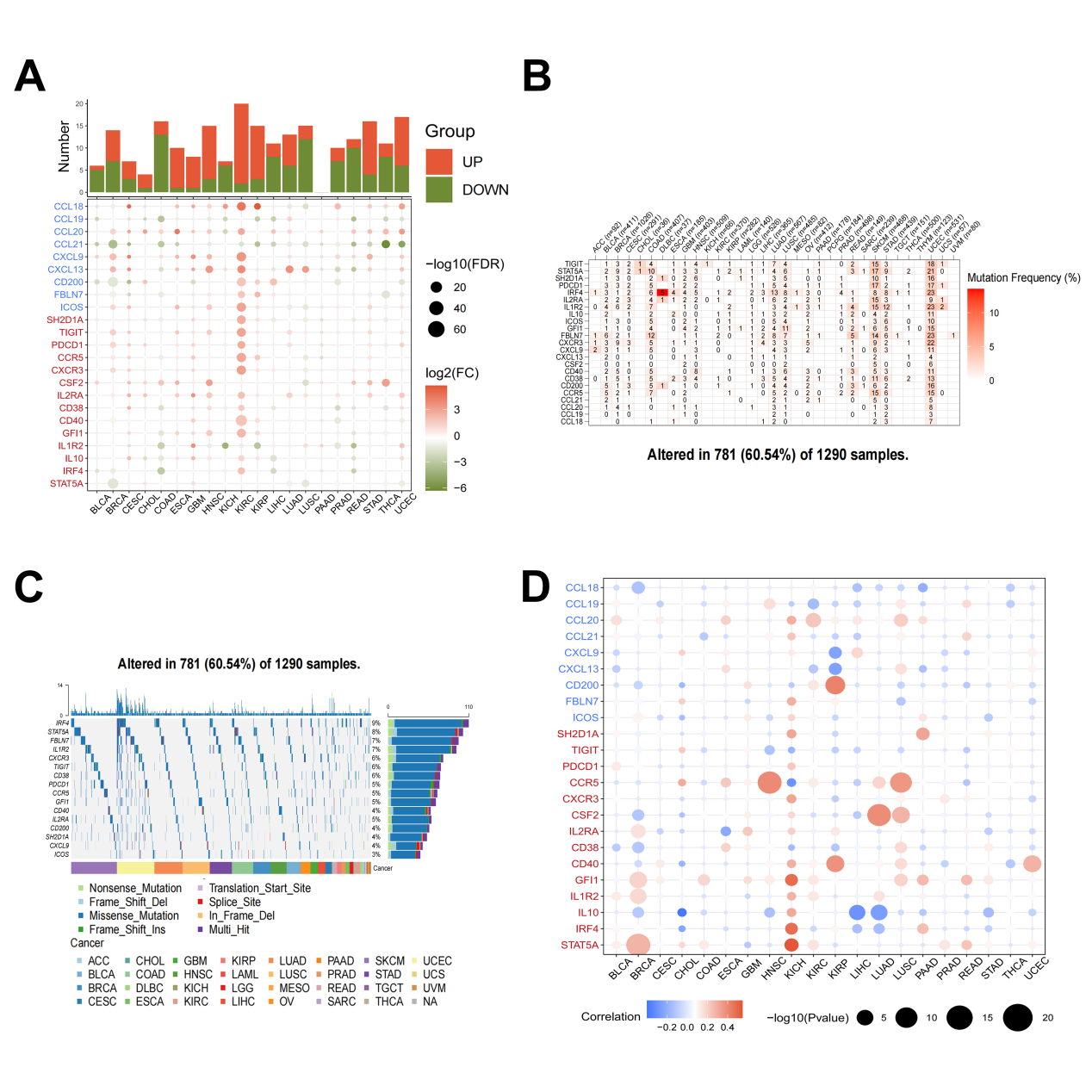
**

**Figure S3. Pan-cancer analysis of TLSRGs expression and mutation patterns.** (A) Pan-cancer analysis on the expression levels of the 23 TLSRGs. (B) Pan-cancer analysis on the CNV of 23 TLSRGs. (C) Pan-cancer analysis on the somatic mutation of the 23 TLSRGs. (D) Pan-cancer analysis on the correlation between somatic mutation and expression levels of the 23 TLSRGs

**
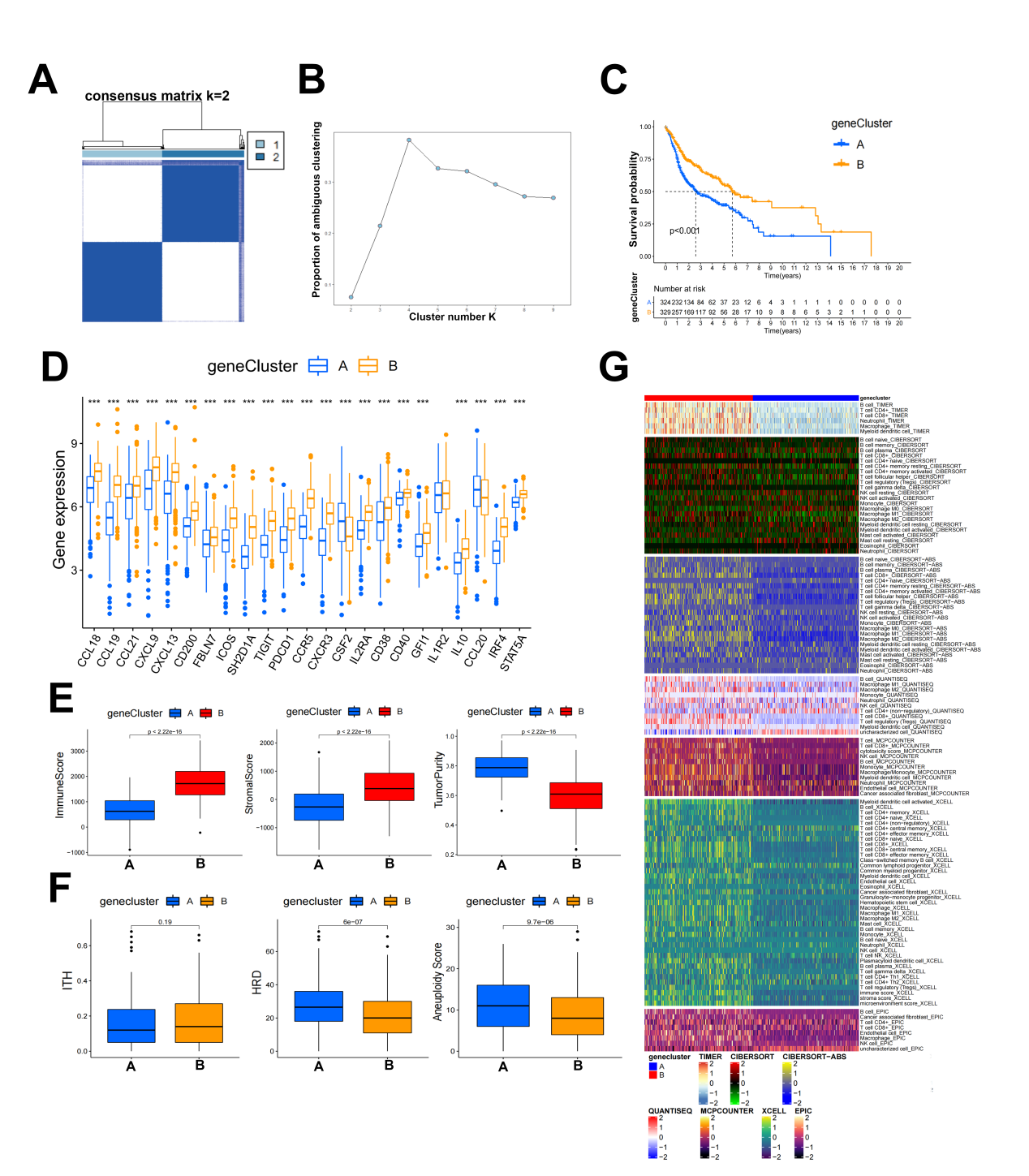
**

**Figure S4. The molecular subtypes of TLSRGs phenotype.** (A) Heatmap of DEGs in two geneClusters identified by unsupervised clustering in consensus matrices for k = 2. (B) Scree plot of cluster numbers ranging from k=2 to 9. (C) KM survival curve for patients in two geneClusters. (D) Gene expression analysis of two geneClusters using ssGSEA method. (E) Comparison of ImmuneScore, StromalScore and tumor purity between two geneClusters using ESTIMATE algorithms. (F) Comparison of HRD, ITH and aneuploidy scores between two geneClusters. (G) Heatmap of immune cell infiltration between two geneClusters using TIMER, CIBERSORT, CIBERSORT-ABS, QUANTISEQ, MCPCOUNTER, XCELL and EPIC algorithms. ^*^*P* < 0.05, ^**^*P* < 0.01, ^***^*P* < 0.001.

**
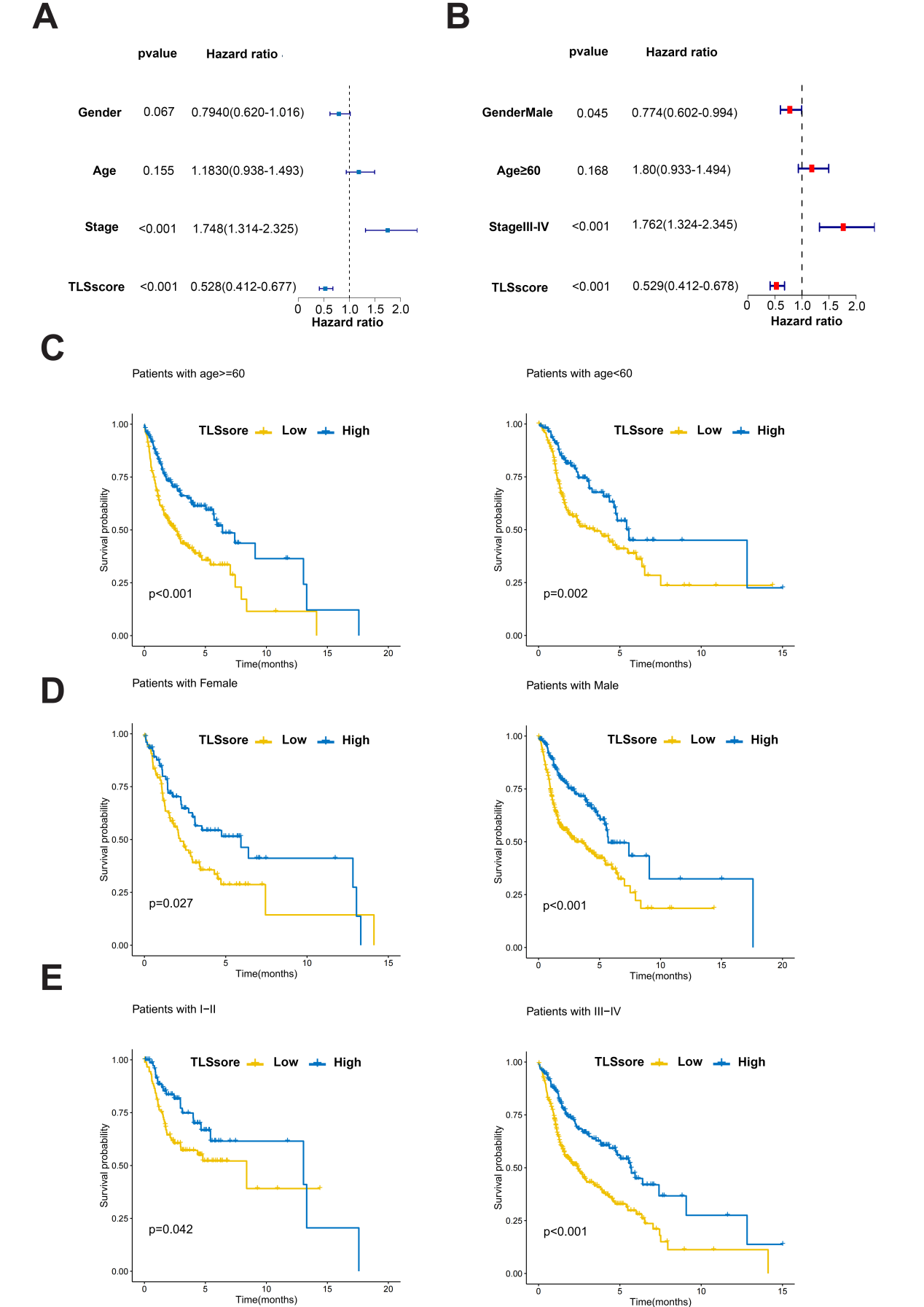
**

**Figure S5. Prognostic analysis of TLSscore and various clinical characteristics.** (A) Univariate Cox regression analysis of clinical parameters. (B) Multivariate Cox regression analysis of clinical parameters. (C) KM survival curve for patients over 60 years and under 60 years in TLSscore^high^ and TLSscore^low^ groups. (D) KM survival curve for female and male patients in TLSscore^high^ and TLSscore^low^ groups. (E) KM survival curve for patients with stage I-II and stage III-IV in TLSscore^high^ and TLSscore^low^ groups.

**
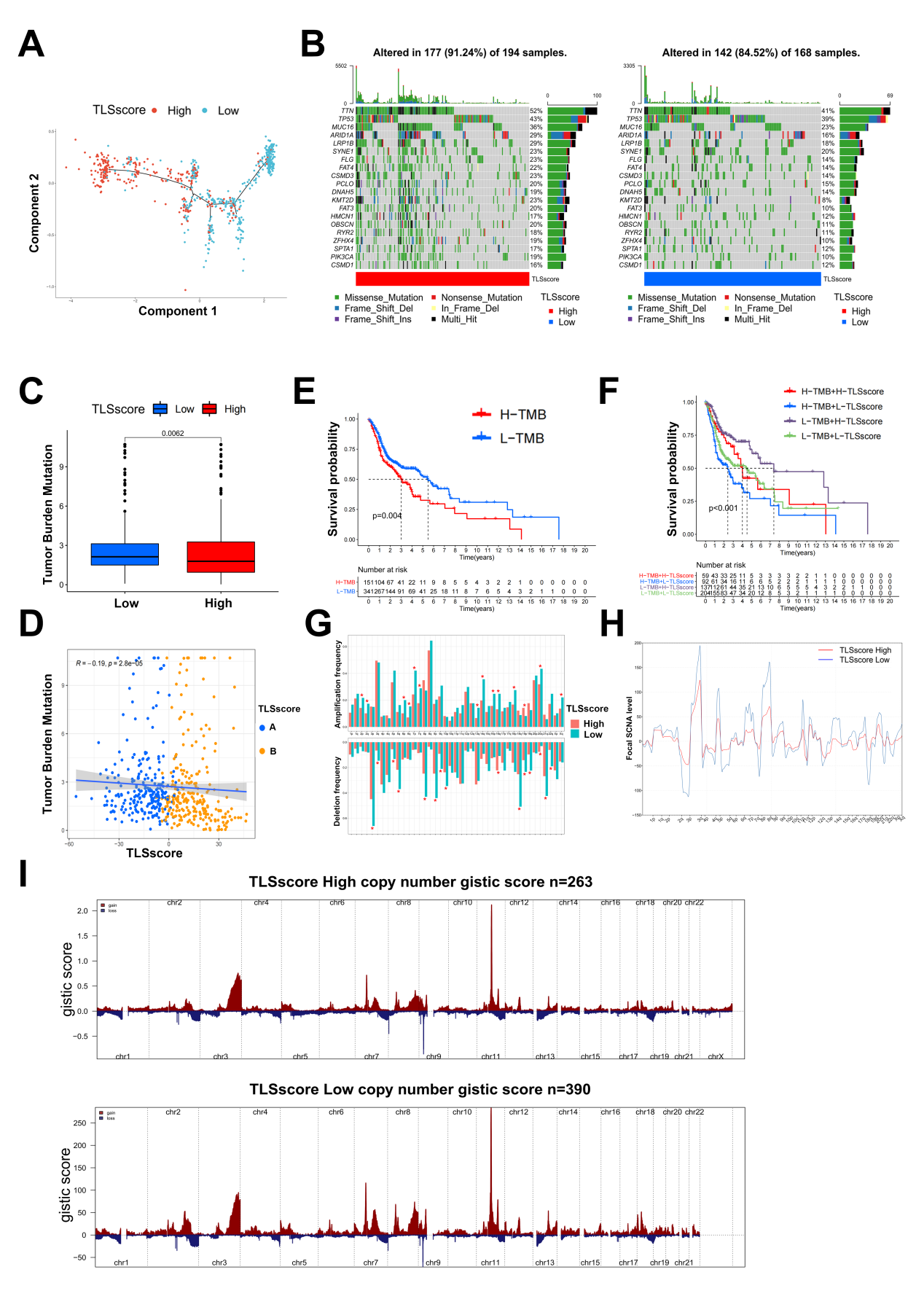
**

**Figure S6. Somatic mutation analysis between high and low TLSscore groups.** (A) Monocle analysis of TLSscore^high^ and TLSscore^low^ groups. (B) The somatic mutation frequency of TLSscore^high^ and TLSscore^low^ groups. (C) The tumor burden mutation of TLSscore^high^ and TLSscore^low^ groups. (D) Linear regression analysis between TLSscore and tumor burden mutation. (E) KM survival curve for patients with high and low tumor burden mutation. H, high; L, low. (F) KM survival curve for patients stratified by tumor burden mutation status and TLSscore. H, high; L, low. (G) CNV analysis of TLSscore^high^ and TLSscore^low^ groups. (H) Focal sCNA analysis of TLSscore^high^ and TLSscore^low^ groups. (I) Manhattan plot illustrating the chromosomal-level of CNV. Upper figure, TLSscore^high^ group, lower figure, TLSscore^low^ groups.

**
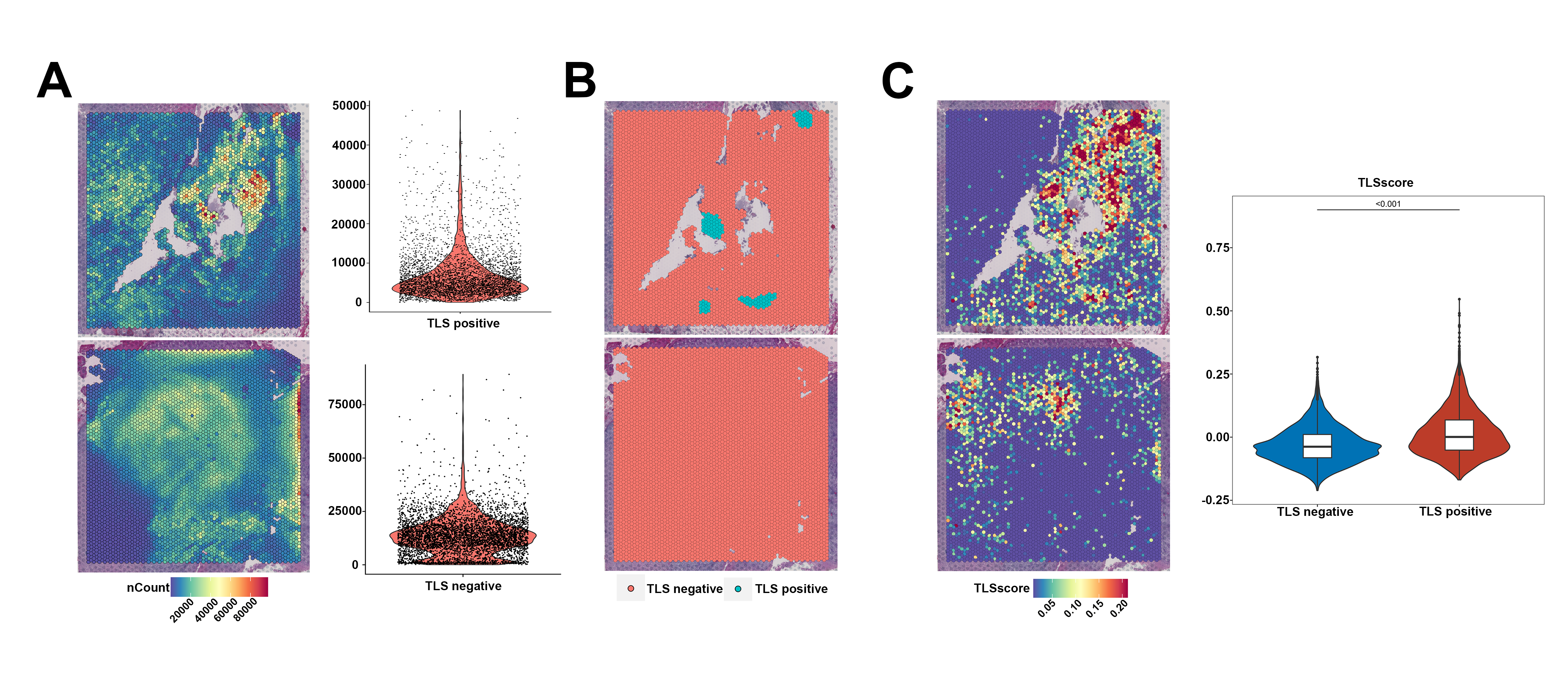
**

**Figure S7. Analysis of TLSscore in HNSCC with TLS at spatial transcriptomics sequencing atlas.** (A) The Normalized Count (nCount) number of each spot in TLS-positive (top left) and negative regions (bottom left). the violin plot represents the respective nCount of TLS-positive (top right) and negative regions (bottom right). (B) The spatial location of TLS in each region. Red spot, TLS negative; blue spot, TLS positive. (C) TLSscore of each spot in TLS-positive (top left) and negative regions (bottom left). The right violin plot represents the comparison of TLSscore between respective TLS-negative/positive groups.

**
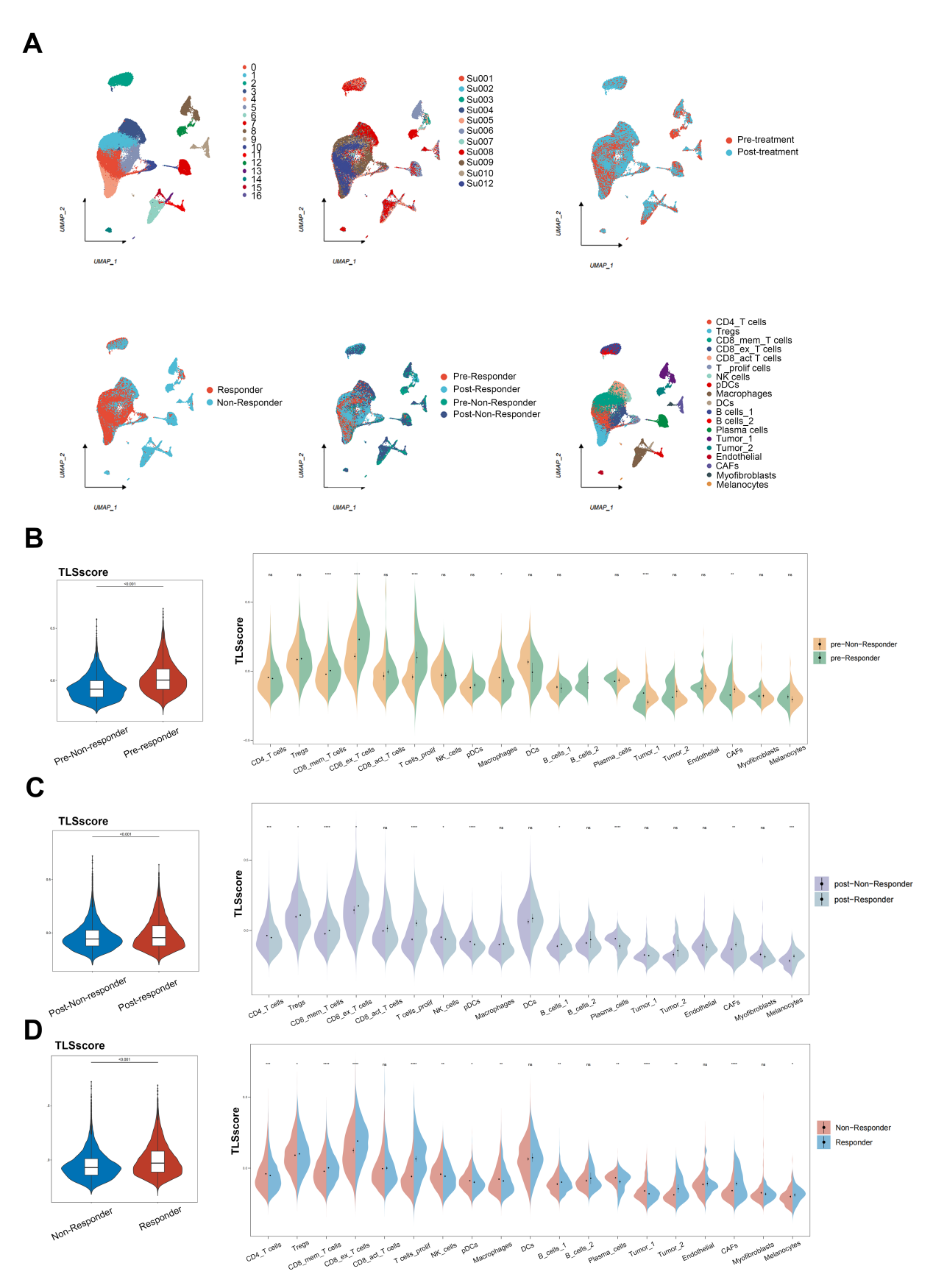
**

**Figure S8. Single cell RNA sequencing analysis for the role of TLSscore in GSE123813.** (A) The UMAP visualization shows the distribution of 17 cell clusters, 11 tumor samples, 19 cell types, patients of responder and non-responder and patients before/after immunotherapy in the GSE123813 dataset. (B) Left panel represents the comparison of TLSscore between pre-responder and pre-non-responder groups in GSE123813; right panel represents the comparison of TLSscore across 19 main cell types between pre-responder and pre-non-responder groups in GSE123813. (C) Left panel represents the comparison of TLSscore between post-responder and post-non-responder groups in GSE123813; right panel represents the comparison of TLSscore across 19 main cell types between post-responder and post-non-responder groups in GSE123813. (D) Left panel represents the comparison of TLSscore between responder and non-responder groups in GSE123813; right panel represents the comparison of TLSscore across 19 main cell types between responder and non-responder groups in GSE123813. ^*^*P* < 0.05, ^**^*P* < 0.01, ^***^*P* < 0.001.

**Supplementary Table**

| Table S1 The clinical characteristics of 3 HNSCC cohorts form TCGA and GEO databases. | | | | |
| --- | --- | --- | --- | --- |
| **Variables** | **Group** | **TCGA Cohort (n=500)** | **GSE42743 Cohort (n=74)** | **GSE41613 Cohort (n=97)** |
| **Age** | **<60** | 221 | 37 | 50 |
|  | **≥60** | 279 | 37 | 47 |
| **Gender** | **Female** | 133 | 16 | 31 |
|  | **Male** | 367 | 58 | 66 |
| **Vital status** | **Alive** | 282 | 32 | 46 |
|  | **Dead** | 218 | 42 | 51 |
| **AJCC Stage** | **I/II** | 107 | 24 | 41 |
|  | **III/IV** | 393 | 50 | 56 |
| **T stage** | **T1** | 46 | 3 |  |
|  | **T2** | 132 | 27 |  |
|  | **T3** | 96 | 28 |  |
|  | **T4** | 171 | 16 |  |
|  | **Tx** | 55 | 0 |  |
| **N stage** | **N0** | 171 | 42 |  |
|  | **N1** | 65 | 13 |  |
|  | **N2** | 164 | 19 |  |
|  | **N3** | 7 | 0 |  |
|  | **Nx** | 93 | 0 |  |
